# An Immunoinformatics Prediction of Novel Multi-Epitope Vaccines Candidate Against Surface Antigens of Nipah Virus

**DOI:** 10.1007/s10989-022-10431-z

**Published:** 2022-06-23

**Authors:** Md. Mahfuzur Rahman, Joynob Akter Puspo, Ahmed Ahsan Adib, Mohammad Enayet Hossain, Mohammad Mamun Alam, Sharmin Sultana, Ariful Islam, John D. Klena, Joel M. Montgomery, Syed M. Satter, Tahmina Shirin, Mohammed Ziaur Rahman

**Affiliations:** 1 Infectious Diseases Division (IDD), icddr,b, 68, Shaheed Tajuddin Ahmed Sarani, Mohakhali, Dhaka 1212 Bangladesh; 2 Institute of Epidemiology, Disease Control and Research (IEDCR), Mohakhali, Dhaka 1212 Bangladesh; 3 EcoHealth Alliance, New York, NY 10001-2320 USA; 4 Viral Special Pathogens Branch, Centers for Disease Control and Prevention, 1600 Clifton Rd. NE, Atlanta, GA 30333 USA

**Keywords:** Nipah virus, Epitope, Subunit vaccine, Immunoinformatics, Simulation, In silico cloning

## Abstract

Nipah virus (NiV) is an emerging zoonotic virus causing outbreaks of encephalitis and respiratory illnesses in humans, with high mortality. NiV is considered endemic in Bangladesh and Southeast Asia. There are no licensed vaccines against NiV. This study aimed at predicting a dual-antigen multi-epitope subunit chimeric vaccine against surface-glycoproteins G and F of NiV. Targeted proteins were subjected to immunoinformatics analyses to predict antigenic B-cell and T-cell epitopes. The proposed vaccine designs were implemented based on the conservancy, population coverage, molecular docking, immune simulations, codon adaptation, secondary mRNA structure, and in-silico cloning. Total 40 T and B-cell epitopes were found to be conserved, antigenic (vaxijen-value > 0.4), non-toxic, non-allergenic, and human non-homologous. Of 12 hypothetical vaccines, two (NiV_BGD_V1 and NiV_BGD_V2) were strongly immunogenic, non-allergenic, and structurally stable. The proposed vaccine candidates show a negative Z-score (− 6.32 and − 6.67) and 83.6% and 89.3% of most rama-favored regions. The molecular docking confirmed the highest affinity of NiV_BGD_V1 and NiV_BGD_V2 with TLR-4 (ΔG = − 30.7) and TLR8 (ΔG = − 20.6), respectively. The vaccine constructs demonstrated increased levels of immunoglobulins and cytokines in humans and could be expressed properly using an adenoviral-based pAdTrack-CMV expression vector. However, more experimental investigations and clinical trials are needed to validate its efficacy and safety.

**Supplementary Information:**

The online version contains supplementary material available at 10.1007/s10989-022-10431-z.

## Introduction

Nipah virus (NiV), is a *Pteropus* bat-borne zoonotic pathogen of the *Henipavirus* genus belonging to the *Paramyxoviridae* family, causing encephalitis and respiratory symptoms in humans in some regions of Asia over the last two decades (Rahman et al. 2013). NiV is a highly contagious virus with a significant public health concern (Wang et al. 2001). It is categorized as a high-priority pathogen by the World Health Organization (WHO) (WHO 2022). NiV is a One Health zoonotic virus that can infect both animals and humans. NiV was first detected in Malaysia and Singapore in 1998–1999 among pig farmers reporting with symptoms of encephalitis. A total of 265 cases were confirmed, including 105 fatalities (Chua et al. 1999; Control and Prevention 1999). Since its discovery, frequent outbreaks have been observed generally between December and March, mainly in Bangladesh and India, with case fatality rates ranging from 70 to 100% (Hsu et al. 2004; Chadha et al. 2006). In Bangladesh, NiV transmission mainly occurs through the consumption of date palm sap contaminated with saliva, urine, and feces of the fruit bats of the genus *Pteropus* (Field 2009; Rahman et al. 2021). Person-to-person transmission has also been documented among family and caregivers of infected NiV patients in several outbreaks (Organization 2004; Sazzad et al. 2013).

NiV is an enveloped, non-segmented, negative-sense RNA virus, displaying surface antigens for attachment to host cell Ephrin B2 and B3 receptors (Vogt et al. 2005; Diederich and Maisner 2007). NiV proteome consists of six structural (N, P, M, F, G, L) and three non-structural (W, V, C) proteins (Wang et al. 2001; Sun et al. 2018). Among those proteins, two surface glycoproteins, G and F proteins, are exposed on the outer surface of the viral envelope. The main function of G protein is to bind the viral particle to the host cell. While G protein facilitates the binding of the virus to the host cell, a conformational change occurs in F protein which mediates the entry of the viral particle into the human cell (Harcourt et al. 2000; Wong et al. 2002; Liu et al. 2015). Several experimental vaccine designs have been proposed or are under development targeting mono-proteins, mainly G protein (Weingartl et al. 2006; Defang et al. 2010; Yoneda et al. 2013; Mire et al. 2013; Ploquin et al. 2013; DeBuysscher et al. 2014; Lo et al. 2014; Prescott et al. 2015), while very few include a multi-protein epitope design incorporating the F and G proteins (Guillaume et al. 2004; Kong et al. 2012; Walpita et al. 2017). Currently, there are no licensed vaccines or drugs available for protection against or treatment of NiV infection in humans or animals. In regions where NiV is endemic, developing a safe and effective vaccine to protect humans and animals against NiV infection is a public and veterinary health priority.

In the context of the recent coronavirus disease (COVID-19) pandemic, most of the commercialized SARS-COV-2 vaccines that are currently available target only one protein (Spike protein) (Salvatori et al. 2020; Malik et al. 2021). During the progression of the pandemic, many variants have emerged mainly due to the mutation in Spike protein, which ultimately creates an issue with vaccine efficacy (Mittal et al. 2022). Furthermore, the high selection pressure of the vaccine targeting only Spike protein in SARS-COV-2 may trigger viral escape mutation by bringing changes in the structure of the selected protein (Moore and Offit 2021). Considering such an issue while designing NiV vaccines, dual antigenic multi-epitope vaccine candidates would be better suited even if naturally occurring mutations happen in any of the targeted NiV-genes. Therefore, this study utilized a combination of antigenic (G and F) proteins to design multi-epitope vaccine candidates. The proposed vaccine may have a lower selective pressure as it will target multiple proteins; simultaneous mutations in G and F proteins will not likely occur at a time. Current research has recently focused on developing multi-epitope vaccines using in silico approaches based on immunoinformatics, eliminating the need to cultivate pathogens and speeding up the vaccine development process (Oany et al. 2014). The multi-epitope vaccines can be a powerful vaccine candidate for clinical trials and have the potential to be effective in the fight against viral infections (Zhang 2018).

In order to develop a vaccine, this study was conducted with the NiV whole-genome sequences available in the NCBI (National Center for Biotechnology Information) database to design epitopes that were conserved in the G and F protein of all available NiV strains to date and contained Cytotoxic T-cell, Helper T-cell, and B-cell epitopes which can trigger immune responses. The growing advances in the field of bioinformatics, in silico design of multi-epitope-based vaccines, have become a powerful tool for vaccine development in the post-genomic era. Therefore, this study was conducted using the various immunoinformatic platforms to propose dual antigenic multi-epitope (DAME) based vaccine designs that can provide additional protective measures for preparedness against larger NiV-outbreaks and pandemics in the future.

## Materials and Methods

**Fig. 1:**
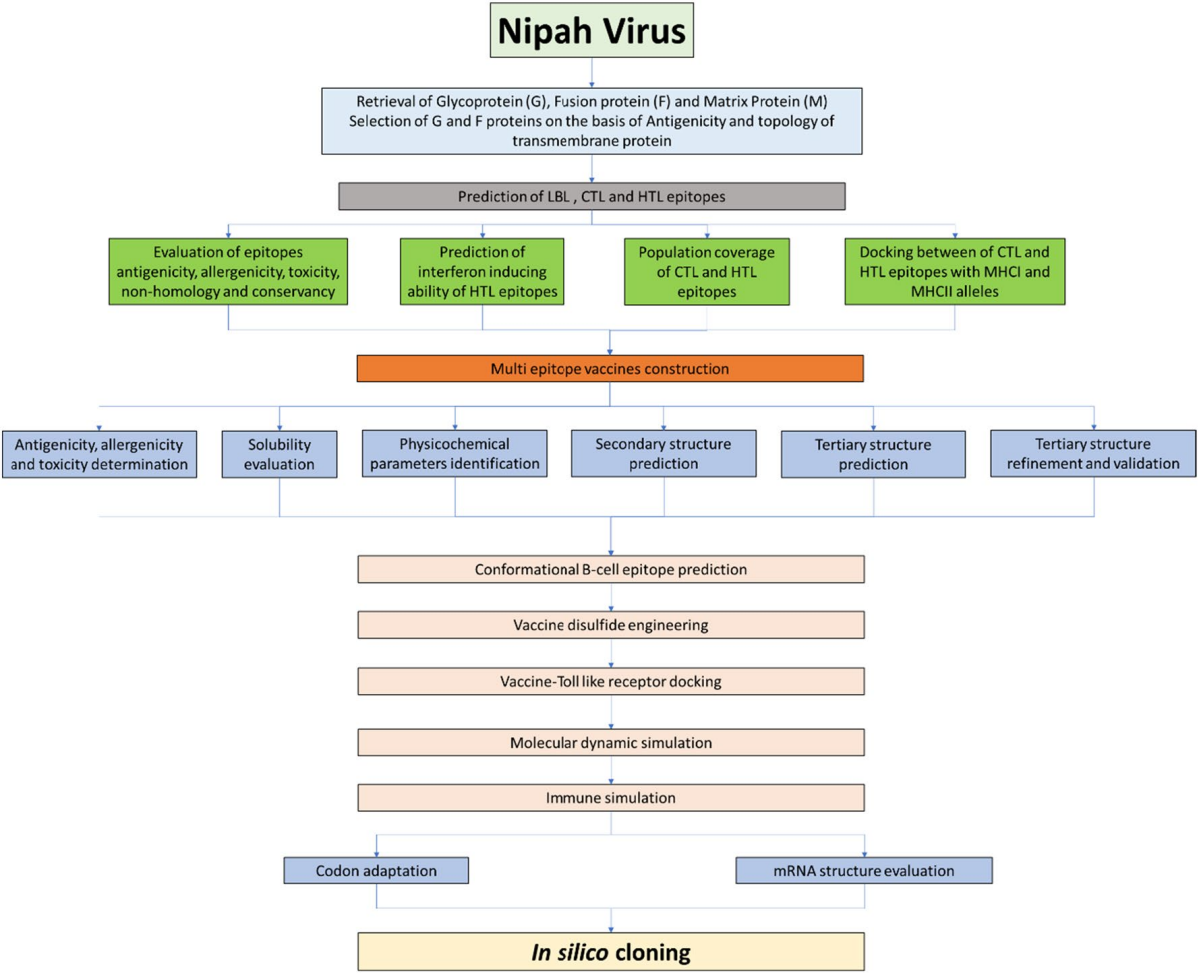
Schematic illustration of overall workflow for developing a vaccine candidate against the Nipah virus

The schematic representation of the experimental procedures performed in our study is summarized below (Fig. 1).

### Sequence Retrieval and Target Protein Selection

A total of 60 high coverage complete genome sequences of NiV available at NCBI (https://www.ncbi.nlm.nih.gov) was retrieved (Table S1). Complete amino acid sequences of G protein (UniProt ID: Q9IH62), F protein (UniProt ID: Q9IH63), and M protein (UniProt ID: Q9IK90) of NiV were retrieved from the UniProtKB (https://www.uniprot.org/help/uniprotkb) database and screened using the Vaxijen 2.0 (http://www.ddg-pharmfac.net/vaxijen/VaxiJen/VaxiJen.html) webserver (Krogh et al. 2001). The threshold value for viral peptide antigenicity was set at 0.4 (Doytchinova and Flower 2007). Glycoprotein-G and matrix protein-M showed significant antigenicity, while the extracellular region of F protein showed to be antigenic. Based on the antigenicity score derived from Vaxijen, G protein was shown to be more antigenic than F protein. TMHMM—2.0 webserver (https://services.healthtech.dtu.dk/service.php?TMHMM-2.0) was used to determine the surface availability of the selected proteins (Doytchinova and Flower 2007). Despite the fact that the full M protein was found to be extracellular by this server, we decided not to use it as a vaccine target since it is surrounded by the viral lipid envelope during the budding process (Wang et al. 2010b), which may result in poor surface availability for immune cell targets. So only the extracellular regions of the F- and G-proteins were selected as vaccine targets.

### Prediction and Screening Linear B-Lymphocyte (LBL), Cytotoxic T-Lymphocyte (CTL), and Helper T-Lymphocyte (HTL) Epitopes

Potential LBL epitopes that can induce the B-cell to elicit antibody production were found using ABCpred (http://crdd.osdd.net/raghava/abcpred/) webserver (Saha and Raghava 2006). The threshold was set at 0.51, and the length for the epitope was set at 16 amino acids. Prediction of CTL epitopes for binding with MHC class I was achieved using the online tool NetCTL-1.2 (Larsen et al. 2007) (https://services.healthtech.dtu.dk/service.php?NetCTL-1.2). A combination of A2-, A3-, B7- and B44-supertypes was considered, resulting in 90% of the population coverage (Sette and Sidney 1999). The threshold value for epitope identification was set at 0.75. Both C terminal cleavage weights and TAP (Transporter associated with antigen processing) transport efficiency weights were set at the default value to provide optimal predictive performance. HTL epitopes prediction for binding with MHC class II was done using the IEDB MHC-II Binding Predictions tool (http://tools.iedb.org/mhcii/) using Consensus 2.22 prediction method (Wang et al. 2010a). The consensus method combines all the available techniques on the server and provides the best possible epitopes. The length of the epitopes was set at 15.

Predicted LBL, CTL, and HTL epitopes were further analyzed to filter out the best epitopes. Toxicity was determined by ToxinPred (Gupta et al. 2013) (http://crdd.osdd.net/raghava/toxinpred/), antigenicity determined by Vaxijen 2.0, allergenicity determined by AllerTOP v.2.0 (https://www.ddg-pharmfac.net/AllerTOP/method.html), and homology was determined by NCBI protein BLAST. Conservancy of G- and F-protein epitopes among previously retrieved 60 NiV (both M and B type) complete genome sequence was determined by IEDB Epitope Conservancy Analysis (http://tools.iedb.org/conservancy/) tool (Bui et al. 2007). Only the epitopes that passed through these filters were further considered. To find out if the predicted HTL epitopes can induce interferon production, they were subjected to IFN-γ prediction webserver IFNepitope (http://crdd.osdd.net/raghava/ifnepitope/) (Dhanda et al. 2013b), IL-4 prediction webserver IL4pred (https://webs.iiitd.edu.in/raghava/il4pred/design.php) (Dhanda et al. 2013a) and IL-10 prediction webserver IL-10Pred (http://crdd.osdd.net/raghava/IL-10pred/) (Nagpal et al. 2017).

### Molecular Docking of the Epitopes

Docking of the epitopes to their respective alleles was performed to determine whether our predicted epitopes can be presented on the cell surface by MHC molecules to elicit the recognition by appropriate T cells. First few highly scored CTL and HTL epitopes were docked against with their representative MHC molecule- HLA-A*02:01 (PDB ID: 4U6Y), HLA-A*03:01 (PDB ID: 6O9C), HLA-B*07:02 (PDB ID: 6VMX), HLA-B*44:02 (PDB ID: 1M60) and HLA-DQA1*01:02 (PDB ID: 6DIG), HLA-DRB1*01:01 (PDB ID: 5V4N), HLA-DRB1*04:01 (PDB ID: 5JLZ) respectively. Extra peptides were eliminated from obtained MHC molecules using PyMol (https://pymol.org/2/), whereas ion, water, and ligand molecules were removed using AutoDock tools before docking. The docking procedure was carried out using the PepDock server (Lee et al. 2015) of GalaxyWEB (https://galaxy.seoklab.org/cgi-bin/submit.cgi?type=PEPDOCK). Finally, the epitope-allele docking score was calculated using the PRODIGY server (https://nestor.science.uu.nl/prodigy/).

### Population Coverage Determination

Due to the polymorphism, MHC molecules can show great diversity among people in different countries or ethnicities, so the approach was to design the vaccine that contains the epitopes that can induce T-cell activity within a broad spectrum MHC allele variation. Population coverage of our selected shortlisted CTL and HTL epitopic alleles was found through the IEDB population coverage tool (http://tools.iedb.org/population/) (Bui et al. 2006). The map showing worldwide population coverage was generated in Rstudio 2022.02.0 using the package rworldmap (South 2011). The code for the construction of the figure can be found at www.github.com/ahsan-adib/Rworldmap-package/blob/main/Rworldmap_PopCov.

### Vaccine Construction and Physiochemical Property Analysis

A total of 12 different models against NiV infections was constructed by applying a different combination of epitopes, adjuvants, and linkers. In short, vaccine constructions were modeled into mainly two ‘Design groups’ based on G-protein epitopes (GPE) and F-protein epitopes (FPE) attachment patterns and position. In Design-1, CTL, HTL, and LBL epitopes have been interlinked by AAY, GPGPG, and KK linkers (Fig. S1-A), respectively, whereas in Design-2, all the GPE followed by FPE was arranged based on their amino acid sequence position and linked through GGGGS linker to form chimeric vaccine (Fig. S1-B). Each of these designs was further classified into 6 models depending on variation in adjuvants (TLR4 agonist (RS09) (Shanmugam et al. 2012), beta-defensin (Q5U7J2) (Mohan et al. 2013), ribosomal protein L7/L12 (P9WHE3.1) (Lee et al. 2014), and the number of Pan HLA DR-binding epitope (PADRE) (Agadjanyan et al. 2005) sequence linked by EAAAK linker. All the vaccine models are given in Supplementary data (Fig. S1).

After construction, these models were then subjected to the various webservers to predict their different properties. Vaxijen 2.0 was used to predict their antigenicity, AllerTOP, and AllergenFP to predict their allergenicity, ToxinPred to predict toxicity, ProteinSol (https://protein-sol.manchester.ac.uk/) to predict solubility, and Protparam to predict stability, thermostability, and hydrophobicity. The instability index and aliphatic index are observed to determine the stability and thermostability of the protein. The grand average of hydropathicity (GRAVY) value was calculated to determine if the antigenic protein is hydrophilic as it is a parameter for easier purification in downstream processing.

### Secondary and Tertiary Structure Prediction of the Vaccine Constructs

The secondary structure of vaccine constructs was determined using PSIPRED (http://bioinf.cs.ucl.ac.uk/psipred/) web tool employing the PSIPRED 4.0 prediction method (McGuffin et al. 2000). PSIPRED uses two feed-forward neural networks based on Position-Specific Iterated—BLAST (PSI-BLAST), which can achieve a Q_3_ score of 81.6%. For validation of the secondary structure, further analysis was carried out using SOPMA (Geourjon and Deleage 1995) secondary structure prediction method (https://npsa-prabi.ibcp.fr/cgi-bin/npsa_automat.pl?page=/NPSA/npsa_sopma.html). Four conformational sites were viewed (Helix, Sheet, turn, and coil), keeping the similarity threshold at 8 and window width at 17. Determination of the tertiary structure of the vaccine models was performed by uploading each of the vaccine candidate sequences to the RaptorX (http://raptorx.uchicago.edu/) online server (López-Blanco et al. 2014). All the predicted models from this server were then imported into Pymol software for visualization.

### Refinement and Validation of the Designed Vaccine Construct

Refinement of the 3D version of the vaccine construct was done through GalaxyRefine web server (https://galaxy.seoklab.org/cgi-bin/submit.cgi?type=REFINE) (Heo et al. 2013; Lee et al. 2016) and then the ProSA web tool (https://prosa.services.came.sbg.ac.at/prosa.php) was used to validate the structure by analyzing the result of the Z score. This score is identified by comparing the uploaded protein to existing proteins containing similar lengths found in the PDB database (Wiederstein and Sippl 2007). To further validate, the PROCHECK (https://www.ebi.ac.uk/thornton-srv/software/PROCHECK/) web tool was employed to determine the stereochemical property of the vaccine construct (Laskowski et al. 1993; Rullmann 1996).

### Immune Simulation and Conformational B-Cell Epitope Prediction

To anticipate humoral and cellular immune responses, cellular entities, and cytokines responses of the designed vaccine candidates, C-immsim webserver (https://kraken.iac.rm.cnr.it/C-IMMSIM) was employed (Rapin et al. 2010; Castiglione et al. 2021). Setting the time steps at 1, 84, and 170 (each time step is 8 h), a total of three injections were given with the interval of 28 days approximately. Simulation steps were set at 1050 to observe immune response for about one year (= 350 days) of window period while all other parameters were set at their default value. The 3D structure of the final vaccine construct can allow different regions of the protein to come in close proximity and thus can act as a discontinuous epitope. To find the possible epitope that can induce B-cell production, the final vaccine construct PDB files were uploaded to the Ellipro server (http://tools.iedb.org/ellipro/) (Ponomarenko et al. 2008). The minimum score was set at 0.5, and the maximum distance was set at 6 Å.

### Protein Di-Sulfide Engineering

DbD2 (http://cptweb.cpt.wayne.edu/DbD2/) webserver was used to design rational disulfide bonds in the protein structure and to determine whether they are consistent from proximity and geometrical perspective (Ponomarenko et al. 2008). As proteins are highly dynamic in nature, mutation can impact the structure and thus alter the protein’s function. The DynaMut webserver (http://biosig.unimelb.edu.au/dynamut/) was employed to predict the change in the entropy due to mutation into cystines and determine whether the mutations in the protein will affect structure stability (Rodrigues et al. 2018).

### Molecular Docking and Dynamic Simulation with TLRs

The ClusPro webserver (https://cluspro.bu.edu/login.php) was used to determine the docking of the vaccine candidates with the Toll-like receptors, TLR2 (PDB ID:6NIG), TLR3 (PDB ID: 7C76), TLR4 (PDB ID: 4G8A), TLR7 (PDB ID: 5GMG), TLR8 (PDB ID: 6ZJZ) and TLR9 (PDB ID: 3WPF) to find out whether the designed vaccine candidates are appropriate to enhance the immune response (Kozakov et al. 2013; Kozakov et al. 2017; Vajda et al. 2017; Desta et al. 2020). TLRs structures were retrieved from Protein Data Bank, and AutoDock tools followed by PyMol were used to remove any ambiguity from the complex form of TLRs. The PRODIGY (PROtein binDIng enerGY prediction) (https://nestor.science.uu.nl/prodigy/) webserver was used to estimate the binding affinities of the docked vaccine-TLRs complexes. Finally, complexes with the highest binding affinities were subjected to the iMod (http://imods.chaconlab.org/) webserver to evaluate the stability and physical movements of the receptor-binding complex (López-Blanco et al. 2014).

### In Silico Expression and Cloning of the Vaccine Candidates into an Adenoviral Based Vector

Firstly, codon optimization was performed using the Java Codon Adaptation Tool server (JCat) (http://www.jcat.de/) to express the vaccine candidates into the *Homo sapiens* expression system (Grote et al. 2005). Two restriction enzymes cleavage sites (*Bgl*II and *Eco*RV) were avoided. After that, the RNAfold (http://rna.tbi.univie.ac.at/cgi-bin/RNAWebSuite/RNAfold.cgi) webserver was employed to predict the thermostability of the mRNA secondary structure of the chimeric vaccine (Lorenz et al. 2011). At the N-terminus of the modeled vaccine, the *Bgl*II restriction site followed by the Kozak sequence was added, whereas, at the C-terminus, the stop codon (TAA) followed by the EcoRV restriction site was applied (Khan et al. 2021). This final construct was then inserted into the pAd-Track-CMV shuttle vector through SnapGene software (from Insightful Science; available at snapgene.com). Insertion of the region was designed between *Bgl*II and *Eco*RV restriction sites under strong CMV promoters.

## Results

### Selection of Target PROTEINS for Vaccine Design

**Fig. 2:**
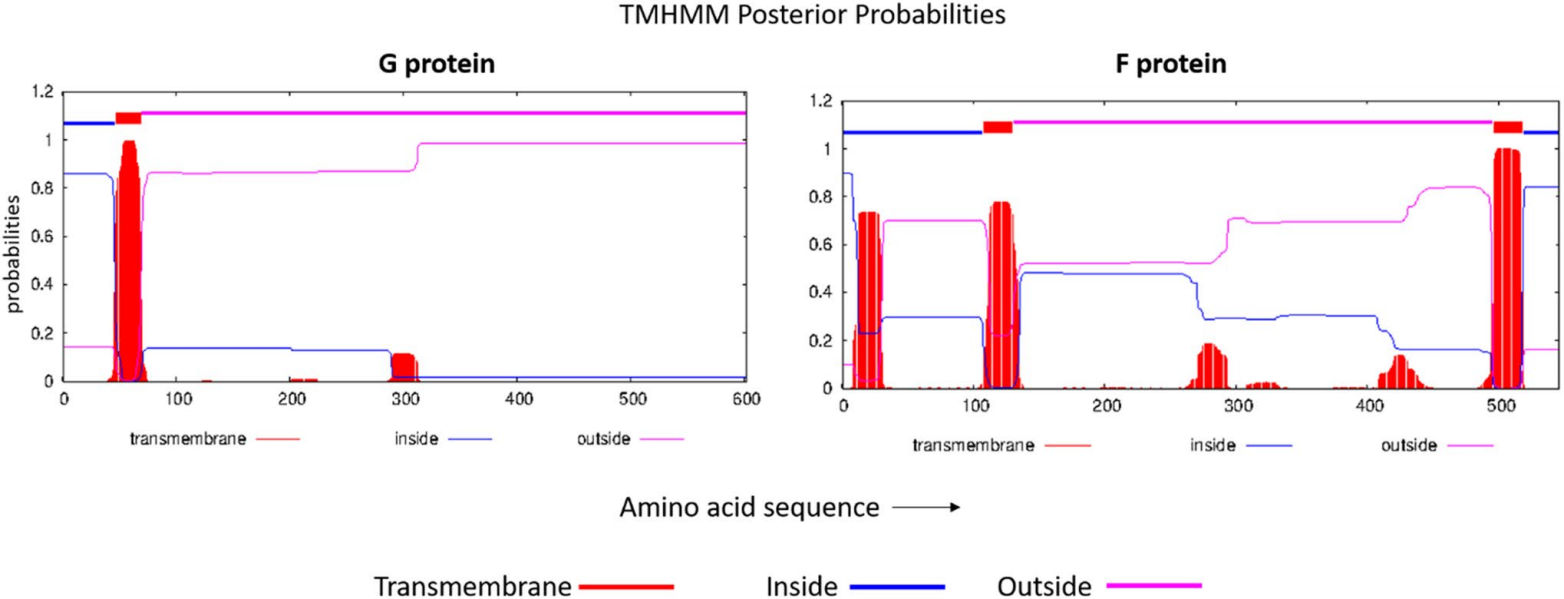
Results of surface availability of G and F protein from TMHMM—2.0 web tool. Purple-colored bar over the amino acid sequences marks the region outside the viral membrane

Physiochemical results identified by the Protparam web tool showed both G and F protein to be thermostable and hydrophilic. Moreover, both proteins were found to be antigenic with values 0.5148 and 0.4688, respectively, estimated by Vaxijen 2.0 webserver. However, we focused our vaccine development on the outer sequence of the viral protein, which was found to be 70–602 amino acid sequence of the G protein and 131–495 amino acid sequence of the F protein (Fig. 2), as determined by the TMHMM—2.0 webserver.

### Prediction and Screening of B-Cell and T-Cell Epitopes

Initially, the ABCpred website predicted 61 and 58 Linear B Lymphocyte (LBL) epitopes for G proteins and F proteins with scores greater than 0.51, indicative of active B cell immune response. Moreover, 60 G proteins epitopes (GPE) and 38 F proteins epitopes (FPE) classified as Cytotoxic T-Lymphocyte (CTL) epitopes were primarily found using NetCTL 1.2 webserver with a combined score not less than 0.75. These CTL epitopes are found to be bound with four (− A2, − A3, − B7 and − B44) major supertypes MHC alleles which contain HLA super motifs that can cover a wide range of HLA types including HLA-A*02:01, HLA-A*68:02, HLA-A*69:01, HLA-A*03:01, HLA-A*11:01, HLA-B*07:02, HLA-B*35:01, HLA-B*37:01, HLA-B*44:02 and HLA-B*44:03 (Table S2-A) (Sette and Sidney 1999). These epitopes with an antigenic score greater than 0.4, non-toxic, non-homologous, and non-allergenic properties, were further evaluated. Considering the conserved epitopic properties for NiV-M (NiV-Malaysia) and NiV-B (NiV-Bangladesh) strains (n = 60 with 100% conservancy) and non-homologous to the human protein (Table 1), 10 GPE and 5 FPE for LBL epitopes, and 10 GPE and 7 FPE as CTL epitopes were subjected to immunoinformatics analysis for possible vaccine designs. Only 8 (3 GPE and 5 FPE) 15-mer Helper T-Lymphocyte (HTL) inducing epitopes were selected after passing the same screening criteria for CTL and LBL, that showed interaction with many different and common MHC-II alleles. Moreover, selected HTL epitopes were able to induce IL-4, IL-10 and and IFN-γ cytokines (Table S2-B).

**Table 1:** Predicted linear B-lymphocyte (LBL), Cytotoxic T-lymphocyte (CTL), and Helper T-lymphocyte (HTL) epitopes of Glycoprotein and Fusion protein for multi-epitope vaccine construction

Epitope	Protein	Epitope Sequence	Start position	Combined Score	Toxicity	Allergenicity	Antigenicity Score	Homology	Conservancy (%)
Predicted B-cell epitopes	G Protein	SKPENCRLSMGIRPNS	390	0.84	No	No	0.4339	NH	100.00
		INWISAGVFLDSNQTA	517	0.83	No	No	0.7413	NH	100.00
		YRAQLASEDTNAQKTI	547	0.80	No	No	0.5336	NH	100.00
		KQRIIGVGEVLDRGDE	246	0.73	No	No	0.9703	NH	100.00
		IGTEIGPKVSLIDTSS	101	0.73	No	No	1.0195	NH	100.00
		PVFYQASFSWDTMIKF	451	0.72	No	No	0.5122	NH	100.00
		PLLAMDEGYFAYSHLE	220	0.70	No	No	0.7393	NH	100.00
		SSTITIPANIGLLGSK	115	0.67	No	No	1.0625	NH	100.00
		PLKIHECNISCPNPLP	152	0.65	No	No	0.4023	NH	100.00
		SNLVGLPNNICLQKTS	179	0.52	No	No	0.5994	NH	100.00
	F Protein	QSGEQTLLMIDNTTCP	403	0.86	No	No	0.5174	NH	100.00
		YIQELLPVSFNNDNSE	292	0.85	No	No	0.5623	NH	100.00
		SEWISIVPNFILVRNT	306	0.70	No	No	0.6820	NH	100.00
		YVLTALQDYINTNLVP	170	0.69	No	No	0.4146	NH	100.00
		ISCKQTELSLDLALSK	190	0.64	No	No	1.4105	NH	100.00
Predicted CTL epitopes	G protein	SLIDTSSTI	110	1.12	No	No	0.6210	NH	100.00
		SLMMTRLAV	313	1.03	No	No	0.7233	NH	100.00
		ITIPANIGL	118	0.79	No	No	1.1090	NH	100.00
		YFPAVGFLV	363	0.77	No	No	0.8129	NH	100.00
		RLSIGSPSK	435	1.56	No	No	0.7713	NH	100.00
		MTRLAVKPK	316	0.78	No	No	1.6597	NH	100.00
		QPVFYQASF	450	1.44	No	No	0.4601	NH	100.00
		KPKLISYTL	199	1.42	No	No	1.0819	NH	100.00
		RPKLFAVKI	589	1.29	No	No	0.5410	NH	100.00
		TEIGPKVSL	103	1.58	No	No	1.4043	NH	100.00
	F protein	LLDTVNPSL	480	1.36	No	No	0.5529	NH	100.00
		SLISMLSMI	487	1.09	No	No	0.4599	NH	100.00
		SIVPNFILV	310	1.07	No	No	0.5759	NH	100.00
		FILVRNTLI	315	1.02	No	No	0.5200	NH	100.00
		KTVYVLTAL	167	0.82	No	No	0.4890	NH	100.00
		TELSLDLAL	195	1.56	No	No	1.1768	NH	100.00
		IEIGFCLIT	326	0.80	No	No	1.4195	NH	100.00
Predicted HTL epitopes	G protein	DAFLIDRINWISAGV	510	NA	No	No	0.9492	NH	100.00
		GVYNDAFLIDRINWI	506	NA	No	No	0.5144	NH	100.00
		VYNDAFLIDRINWIS	507	NA	No	No	0.5724	NH	100.00
	F protein	DPVSNSMTIQAISQA	220	NA	No	No	0.5392	NH	100.00
		ISIVPNFILVRNTLI	309	NA	No	No	0.6730	NH	100.00
		PNFILVRNTLISNIE	313	NA	No	No	0.6480	NH	100.00
		YYIIVRVYFPILTEI	274	NA	No	No	1.0359	NH	100.00
		IGFCLITKRSVICNQ	328	NA	No	No	1.4667	NH	100.00

*NH non-homologous, *NA* not applicable

### Molecular Docking of CTL and HTL Epitopes with HLA Alleles

**Fig. 3:**
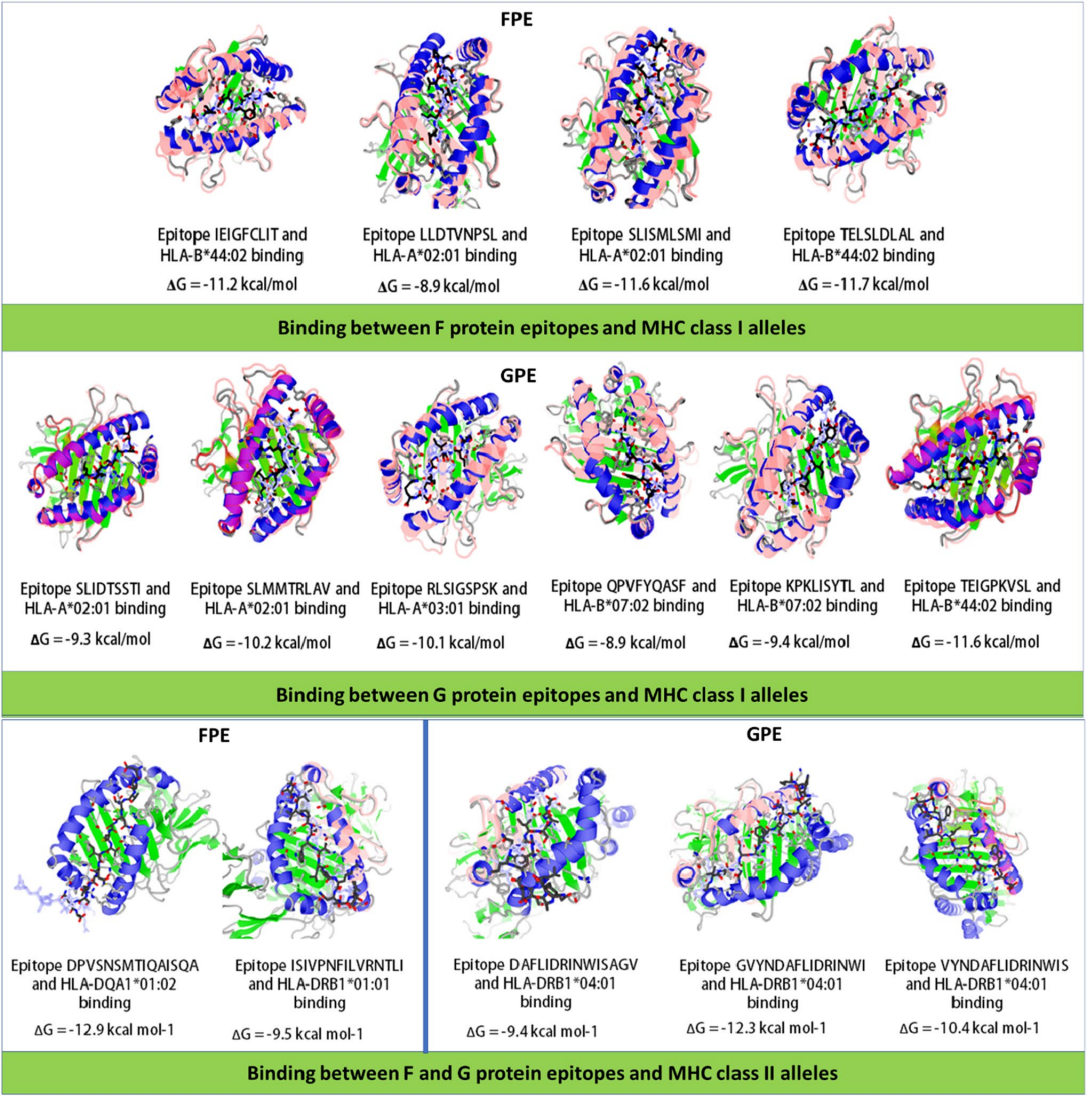
Molecular docking of F protein epitopes (FPE) and G protein epitopes (GPE) with respective HLA alleles. Top representative epitopes were taken for each protein and their binding was shown with alleles with the highest affinity. Protein-peptide docking was performed using GalaxyPepDock server. Free energy (ΔG) value of each binding shows the affinity between epitopes and alleles and was determined through PRODIGY server. Here, ribbon structures denote HLA alleles whereas ball and stick structures represent the epitopes

Docking between top-scored epitopes with their respective HLA alleles was observed to determine the effective binding that can further induce helper T-cell activity. Docking using ‘GalaxyPepDock’ server revealed highly favored molecular interaction between FPE, GPE with HLA alleles (Fig. 3). Negative binding affinities (ΔG ≤  − 8.9 kcal/mol, average =  − 10.5 kcal/mol) were identified by the PRODIGY webserver, proving that the bindings were thermodynamically stable and predicting a strong CTL and HTL response.

### Population Coverage Analysis

**Fig. 4:**
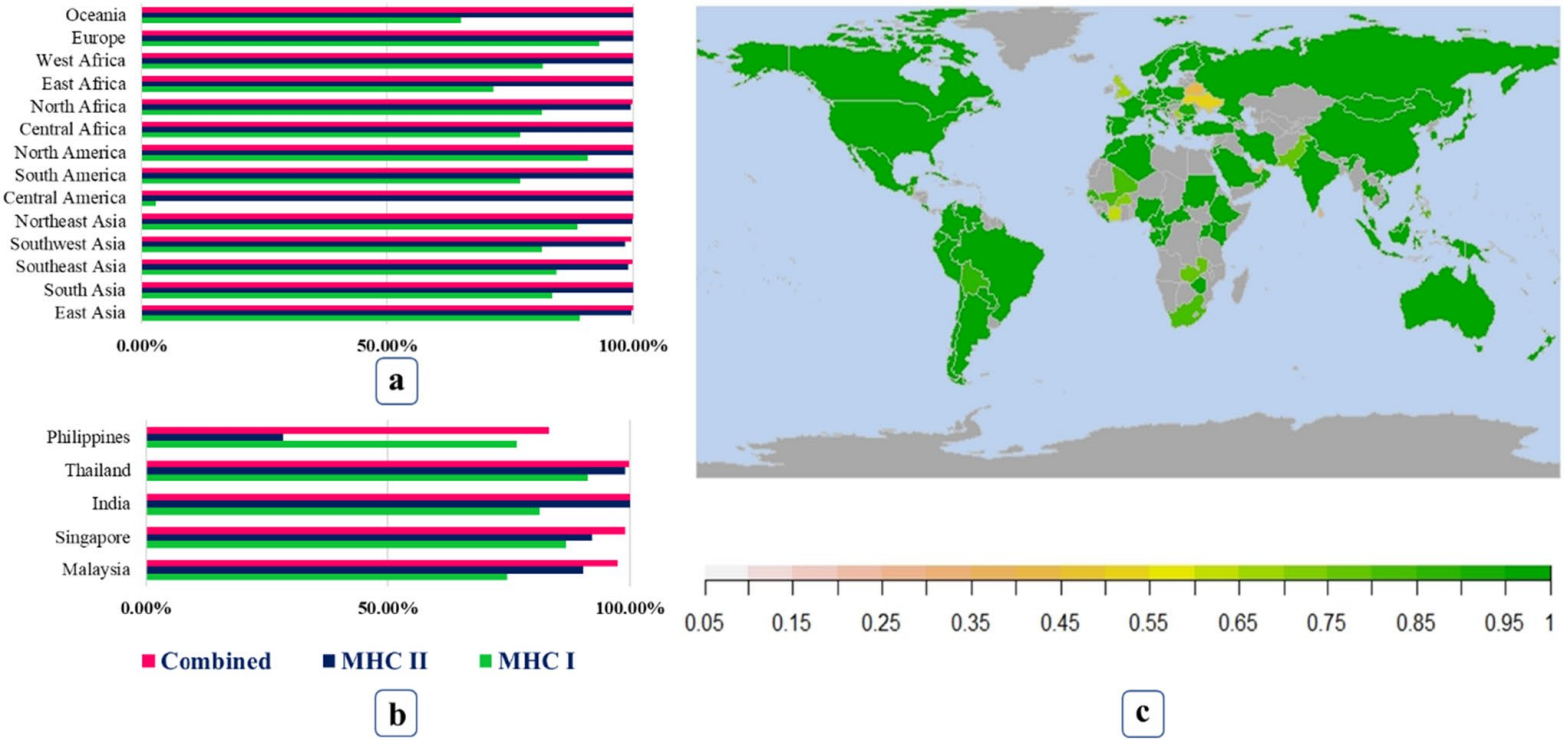
Population coverage of the selected HTL and CTL epitopes and their respective alleles. Bar plots illustrates the population coverage of the epitopes both combinedly and individually (Either MHC-I or MHC-II). **a** Population coverage in a different region of the world, **b** Epitope coverage in areas where previously Nipah outbreaks were observed, **c** World map indicating population coverage in different countries, here gray-colored countries indicates unavailability of data

The selected CTL and HTL epitopes covered 88.73% and 99.94% of the global population, respectively (Fig. 4). More importantly, when combined with both types of epitopes, the resultant alleles covered 99.99% of the world population. About 18 countries of the world show 100% population coverage, including Sweden, Germany, England, Japan and the United States, based on both CTL and HTL epitopes. In Malaysia, where Nipah has first reported, the population coverage for CTL and HTL found 74.61% and 90.26%, respectively with a combined coverage of 97.53%. In India, the Nipah outbreaks were reported repeatedly showing 81.30% and 99.94% population coverage for CTL and HTL, with a combined coverage of 99.99% (Fig. 4b). However, some of the country-specific data could not be generated due to the unavailability of these data in the respective webserver. Nevertheless, region-specific population data covered the worldwide population as a whole. For example, Bangladesh, which is located in South Asia and marked as a Nipah pandemic area, could show a high population coverage as the population coverage of South Asia is 99.99%, with high CTL and HTL coverage that is 83.60% and 99.94%, respectively (Fig. 4a). In addition to geographical distribution, we found good coverage for ethnic groups. Twenty-two ethnic groups have 100% population coverage out of 156. Moreover, approximately 77.56% (121/156) showed greater than 90% population coverage.

### Vaccine Construction and Properties Identification

Considering all the desired epitopes that can induce CTL, HTL, and B-cell responses, two vaccines were designed; which were further classified into three different models for each design depending on the selected adjuvants. TLR4, Beta-defensin, and ribosomal protein L7/L12 were the chosen 3 adjuvants for a higher level of antigenic response. In total, 12 vaccine constructs were designed depending on adjuvant and linker position (Fig. S1).

The subunit vaccine candidates were further analyzed for antigenicity, toxicity, and allergenicity using the aforementioned webservers. Among the 12 models, three models (Design-1 model 1, Design-2 model 2, and Design-2 model 6) were predicted to show allergenic response upon administration so they were excluded from further investigation (Table S3). The Protein-Sol website indicated that Design-1 models showed more solubility, scoring above the threshold value of 0.4.

### Prediction and Validation of Secondary and Tertiary Structure of the Vaccine Constructs

**Fig. 5:**
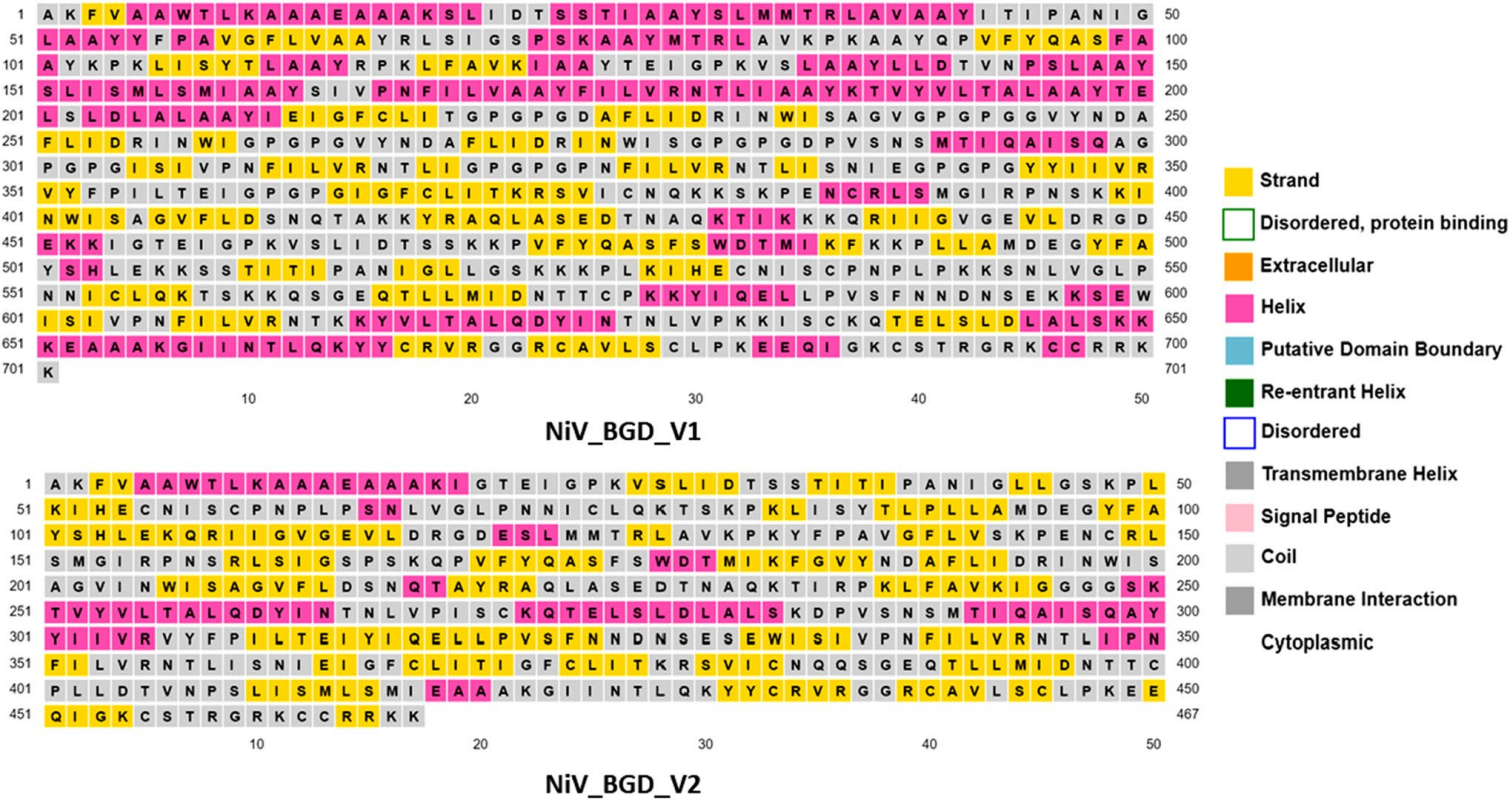
Secondary structure properties of the selected vaccine candidates, NiV_BGD_V1 and NiV_BGD_V2

**Fig. 6:**
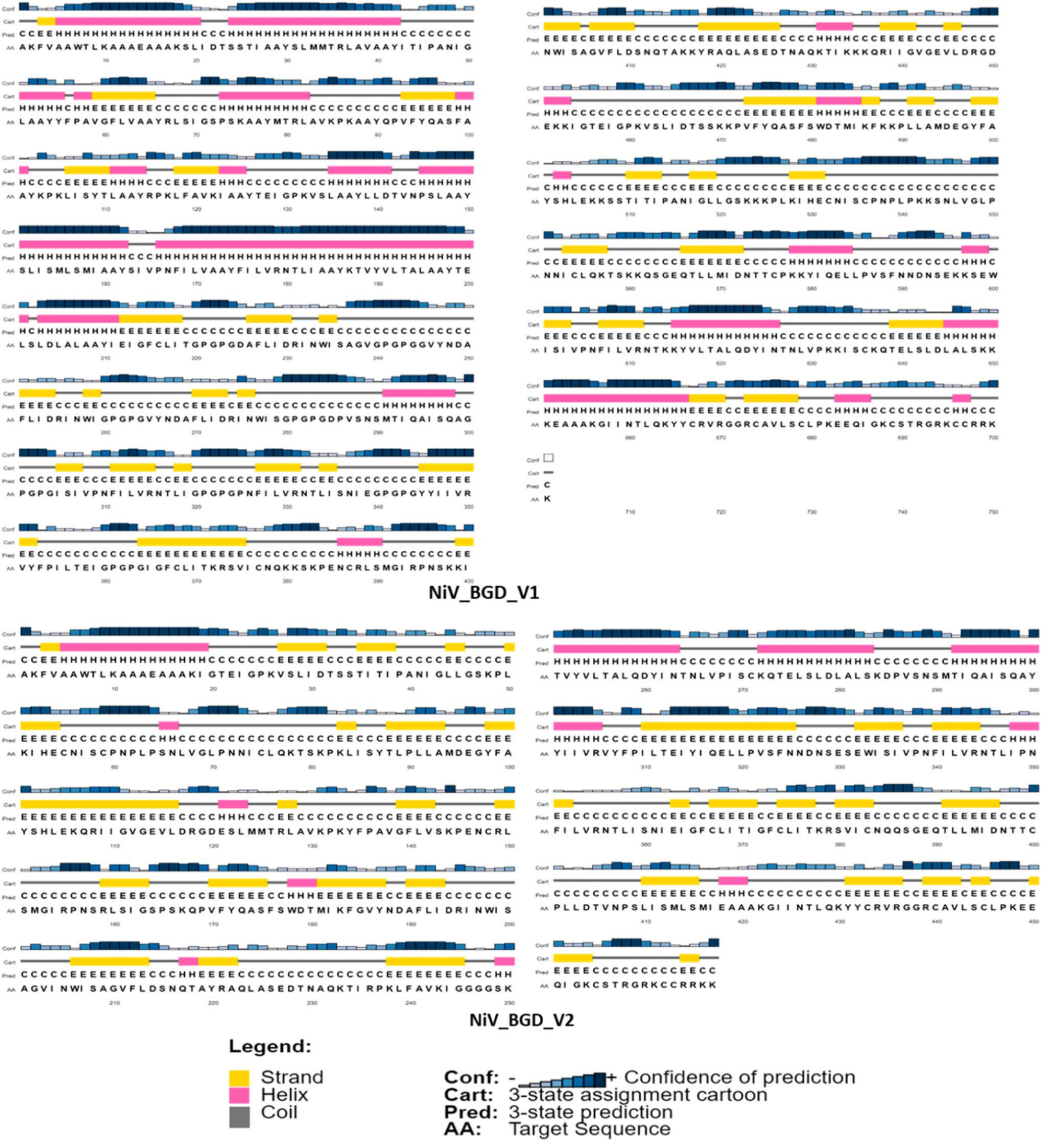
Graphical representation of selected vaccine candidates' secondary structure. NiV_BGD_V1 shows 30.53% α-helix, 27.25% β-sheet and 33.52% coil, and NiV_BGD_V2 shows 19.70% α-helix 31.48% β-sheet, and 41.11% coil

**Fig. 7:**
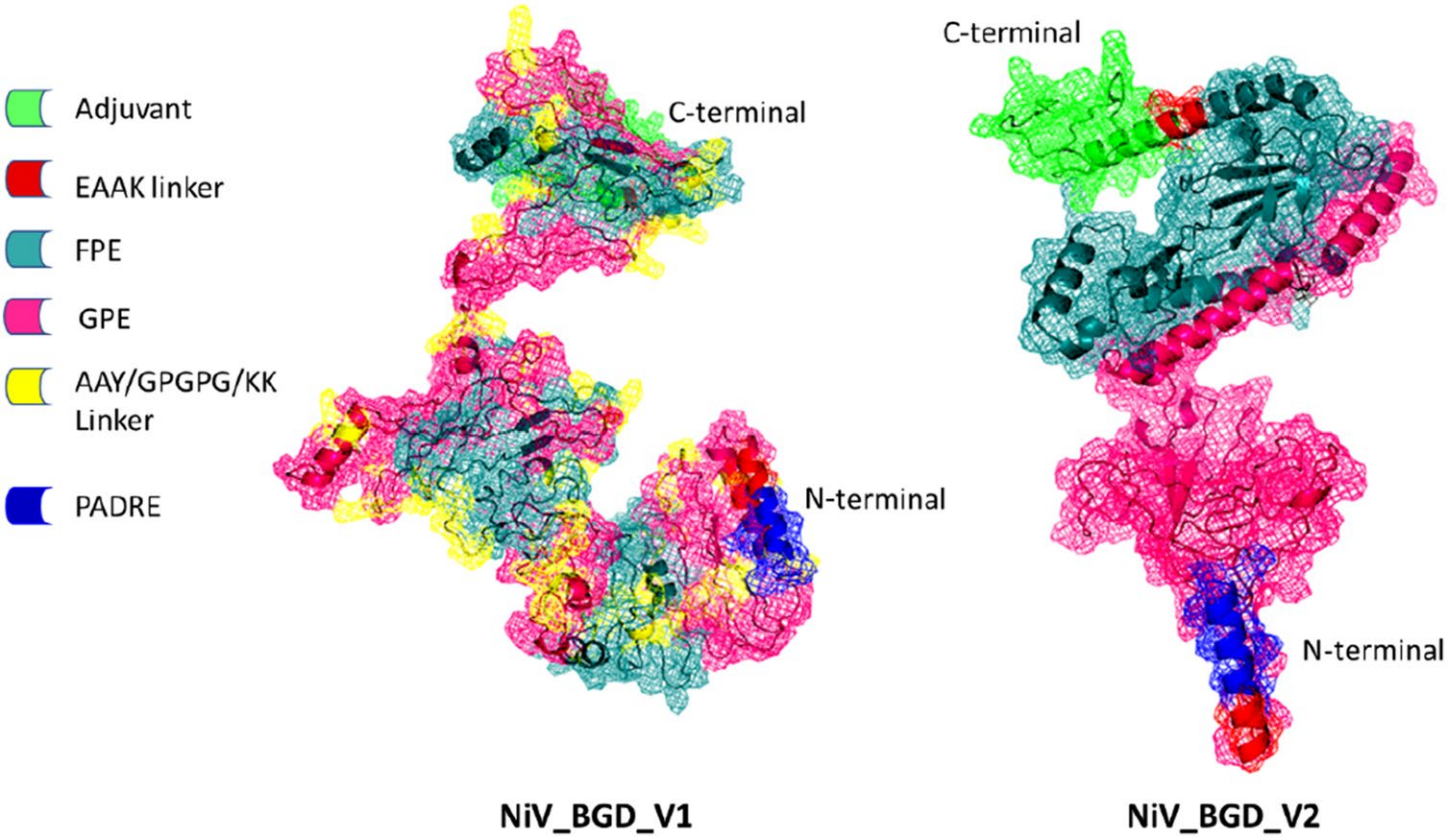
Three-dimensional diagram of the selected vaccine candidates

The remaining nine models were subjected to a PSIPRED webserver to determine the secondary structure of each vaccine construct (Fig. 5). PSIPRED specifically analyzes the regions as a strand, helix, and coil of the given peptide (Fig. 6). Submission of the vaccine constructs’ sequences to the SOPMA webserver shows each of the secondary structure properties (Table S4). It determines the number of comprised peptides. The protein sequence of all nine models was uploaded to the RaptorX webserver to determine the tertiary structures. The webserver analyzed the protein sequences and resulted in a possible 3-dimensional configuration for each construct (Fig. 7). Using the GalaxyRefine web tool, all models were refined and then carefully examined to identify the presence of a gap in the 3D construct. The gap in the 3D structure will result in fragmentation of the protein and will make the protein subunit vaccine invalid/unstable. Of the remaining nine models, five models showed no gap in their 3D structure resulting in a non-fragmented entity and were chosen for further evaluation.

**Fig. 8:**
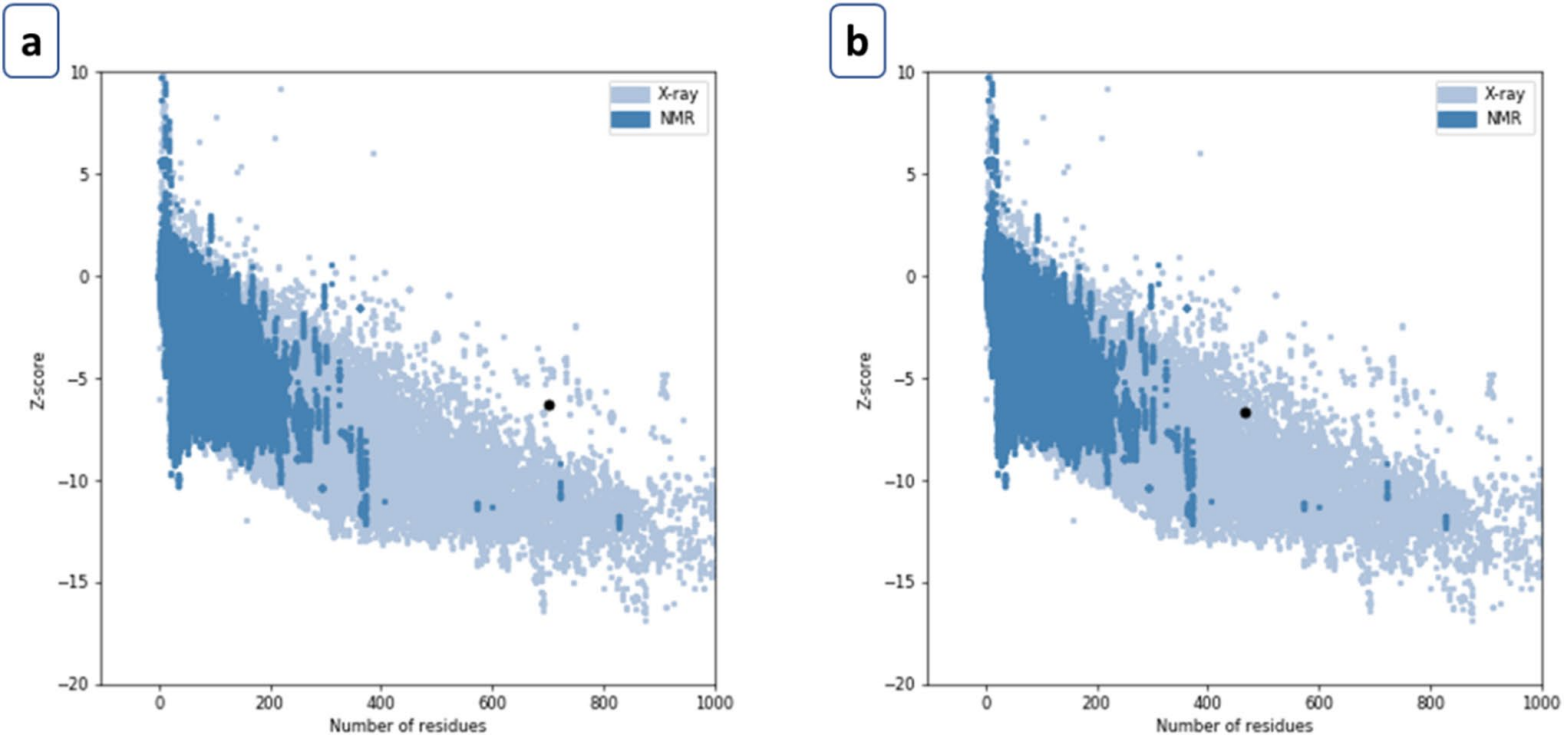
**a** NiV_BGD_V1 model validation resulting Z score of − 6.32. **b** NiV_BGD_V2 model validation resulting Z score of − 6.67

**Fig. 9:**
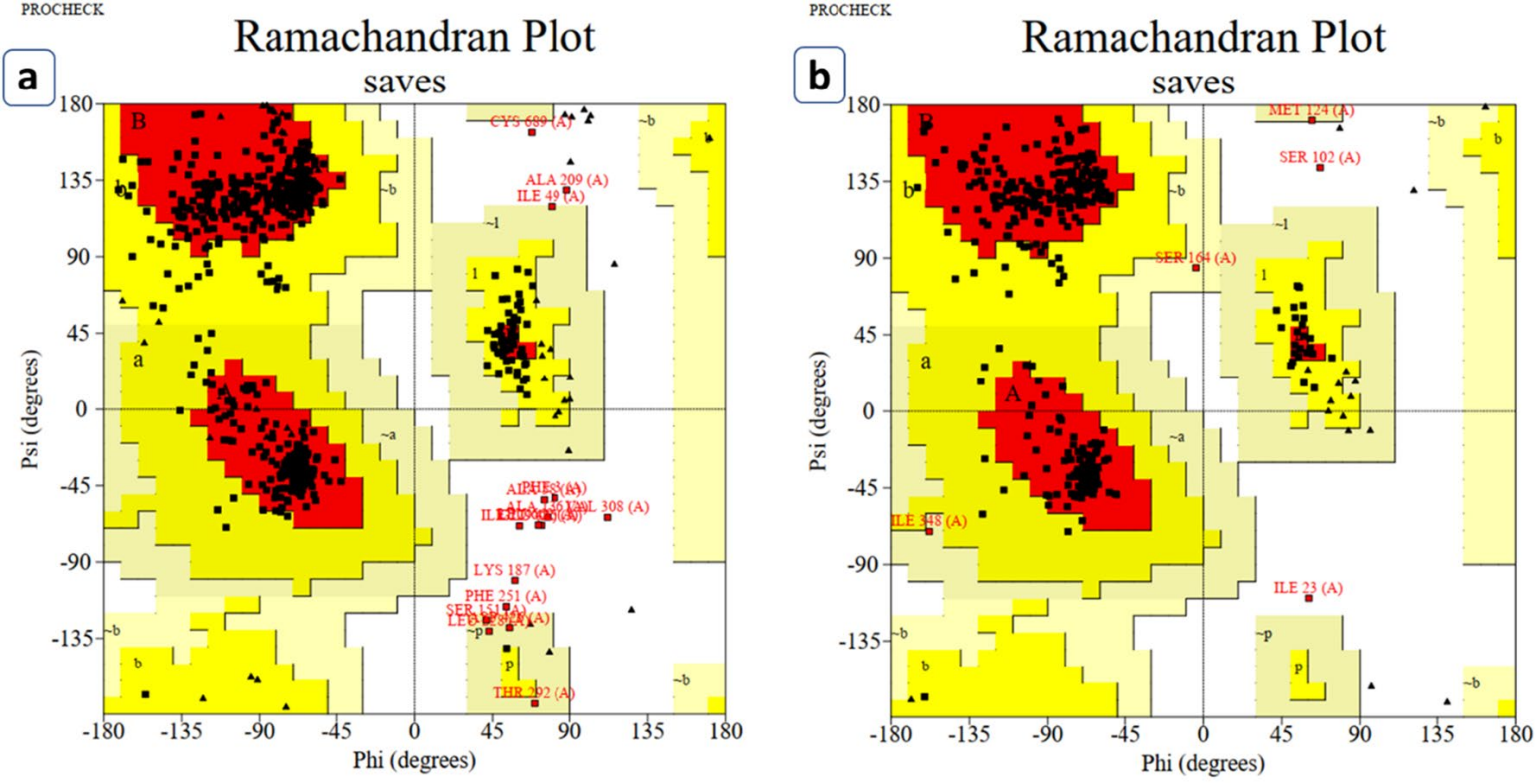
Ramachandran plot analysis of **A** NiV_BGD_V1 and **B** NiV_BGD_V2. (a) NiV_BGD_V1 shows 83.6% in the allowed region and 14.7% and 0.8% in the additional and generously allowed region, respectively. Only 1.8% showed to be in the unallowed region. (b) NiV_BGD_V2 shows 89.3% in the allowed region and 9.4% and 0.7% in the additional and generously allowed region, respectively. Only 0.5% showed to be in the unallowed region

Vaccine models were validated using ProSA and PROCHECK web tools sequentially. At first, the ProSA web tool was used to determine the Z value of each vaccine (Fig. 8) construct to determine energy distribution derived from random conformation (Sippl 1993, 1995). Structure validation shows that the Z score of these two candidates is − 6.32 and − 6.67, respectively. PROCHECK web tool was used to plot the protein region in the Ramachandran plot identify residues of the proteins in the allowed and disallowed region (Fig. 9). Two of the remaining five vaccine constructs contained the highest number of residues in the allowed region. These two model constructs were considered as suitable vaccine candidates and named as NiV_BGD_V1 and NiV_BGD_V2, respectively. Vaccine candidate-1 (NiV_BGD_V1) contained 83.6%, and Vaccine candidate-2 (NiV_BGD_V2) contained 89.3% in the most favored region.

Additionally, physicochemical properties of the selected vaccine candidates predicted NiV_BGD_V1 to be soluble and NiV_BGD_V2 to be insoluble in water, which indicates that the second vaccine candidate to be a single-shot vaccine administered into the body. Other physicochemical parameters of both vaccine candidates were well in range to be considered as a potential vaccine (Table 2).

**Table 2:** Physiochemical Properties of proposed vaccine candidates

SL	Physiochemical properties	Results	
		NiV_BGD_V1	NiV_BGD_V2
1	Number of amino acids	701	467
2	Molecular weight	76,362.62	51,486.21
3	Theoretical pI	9.67	9.14
4	Formula	C_3484_H_5583_N_903_O_965_S_25_	C_2312_H_3734_N_610_O_665_S_24_
5	Instability index	29.35	39.78
6	Aliphatic index	100.4	105.25
7	Grand average of hydropathicity (GRAVY)	0.074	0.133
8	Estimated half-life (mammalian reticulocytes, in vitro)	4.4 h	4.4 h
9	Estimated half-life (yeast, in vivo)	> 20 h	> 20 h
10	Estimated half-life (*Escherichia coli*, in vivo)	> 10 h	> 10 h
11	Extinction coefficients (at 280 nm in water)	90,035 M-1 cm-1	50,725 M-1 cm-1
12	Antigenicity (AntigenPRO)	0.528842	0.624424
13	Antigenicity (Vaxijen)	0.5924	0.6729
14	Allergenicity (AllerTOP)	Non-allergen	Non-allergen
15	Allergenicity (AllergenFP)	Non-allergen	Non-allergen
16	Solubility (proteinSOL) (0.45)	0.424	0.304
17	Solubility upon overexpression (solPRO)	SOLUBLE with probability 0.839197	INSOLUBLE with probability 0.756665
18	Disordered region (%)	1.426533524	2.141327623

### Conformational B-Cell Epitopes Identification

**Fig. 10:**
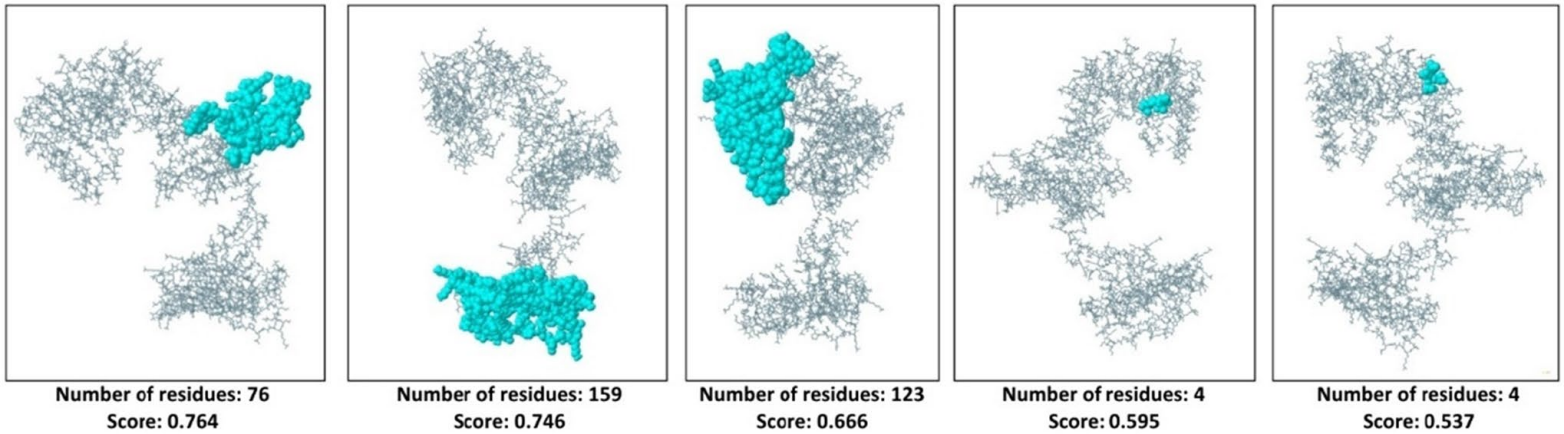
Conformational B-cell epitopes of vaccine candidate-1 (NiV_BGD_V1) that are displayed in the colored region. Length and score from the ElliPro webserver are shown below each epitope

**Fig. 11:**
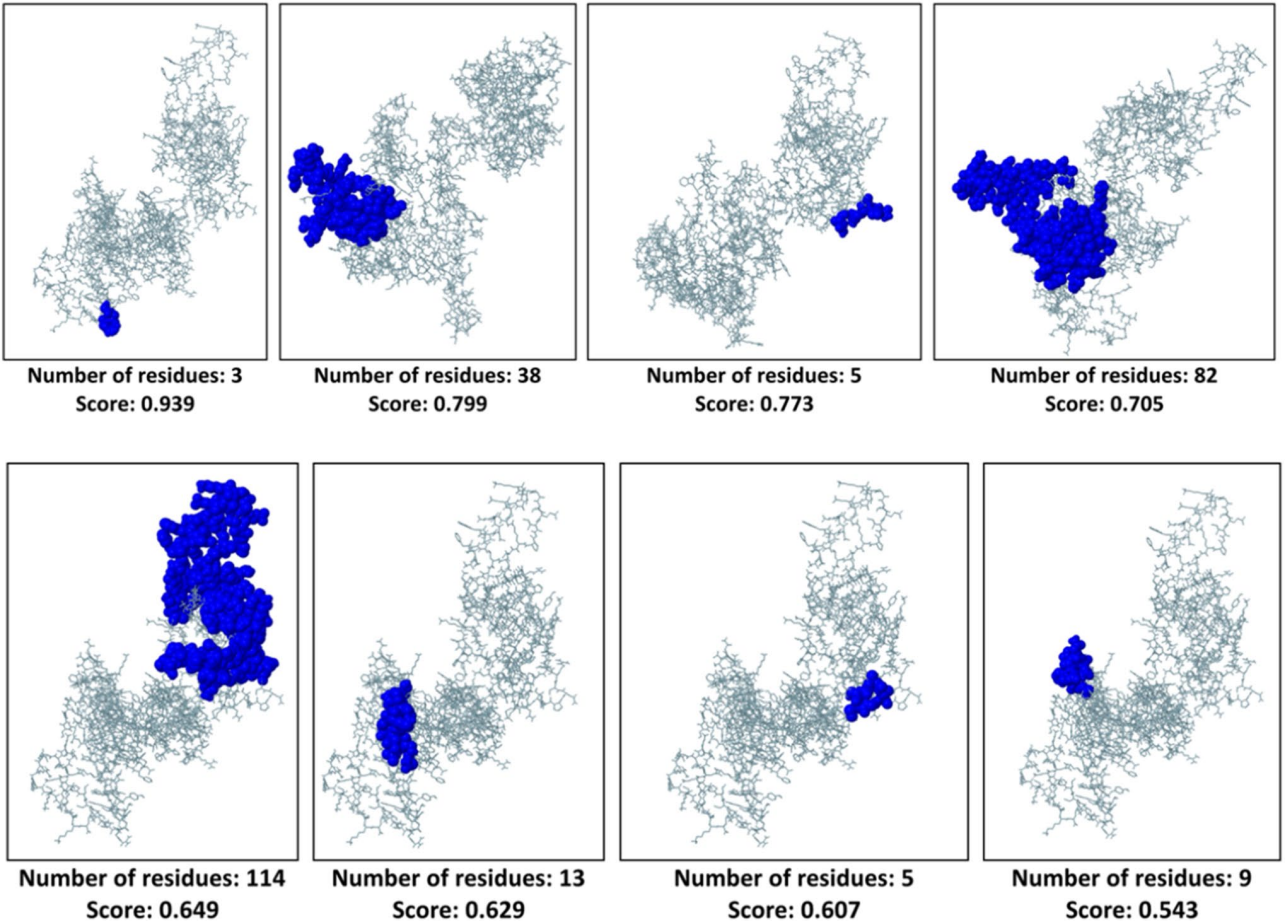
Conformational B-cell epitopes of vaccine candidate-2 (NiV_BGD_V2) that are displayed in the colored region. Length and score from the ElliPro webserver are shown below each epitope

The ElliPro webserver derived three-dimensional conformational epitopes that can induce B-cell activity. NiV_BGD_V1 resulted in 5 epitopes (Fig. 10) while NiV_BGD_V2 showed 8 B-cell epitopes (Fig. 11; Table S5).

### Disulfide Engineering of the Final Vaccine Construct

**Fig. 12:**
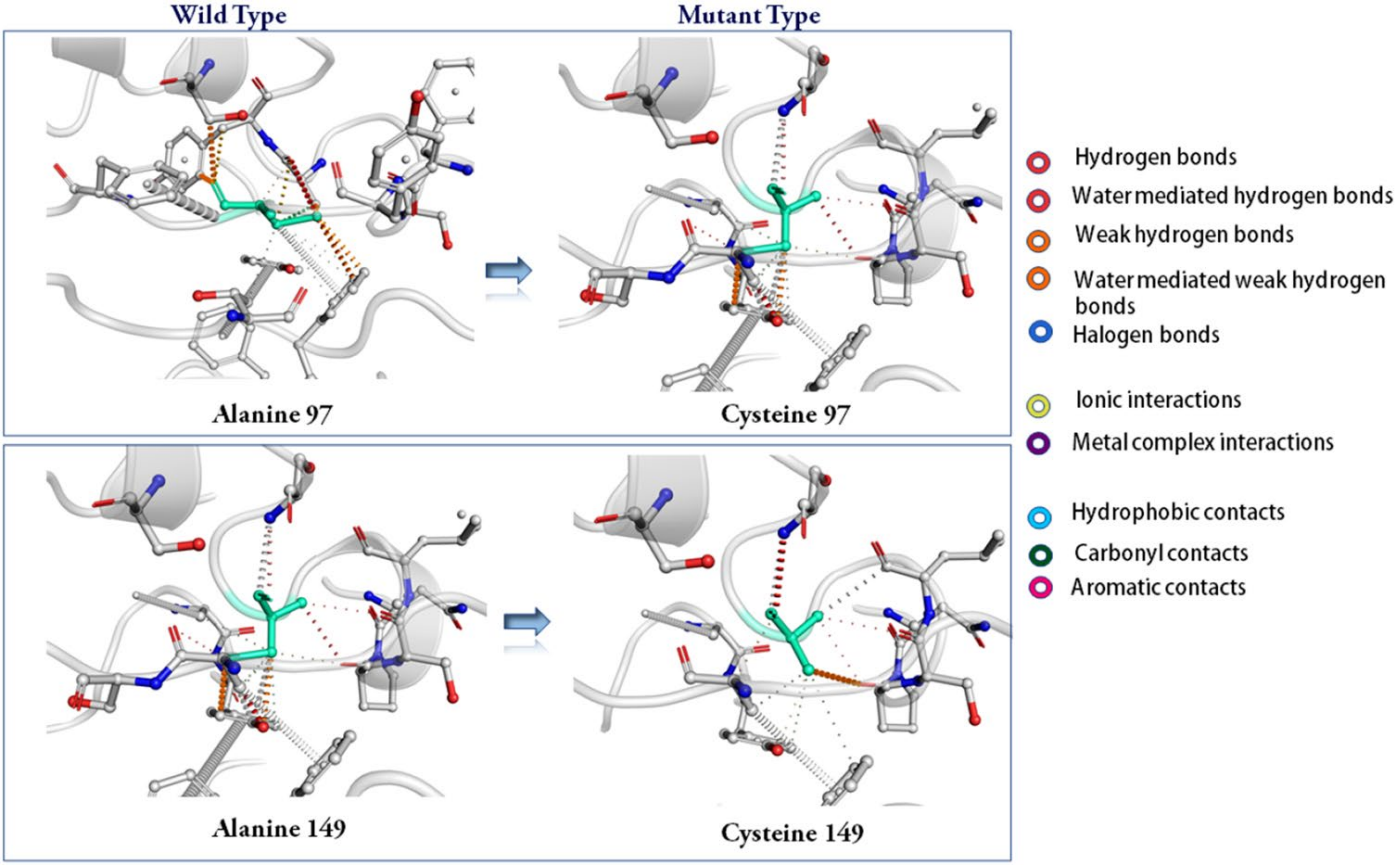
Wild type and mutant type is shown on the protein structure containing Ala97 and Ala149, respectively

Disulfide by Design-2 (DbD2) webserver predicted a total of 56 pairs of residues for NiV_BGD_V1 and 36 pairs of residues for NiV_BGD_V2 for the probable formation of disulfide bonds. Among the selected pairs, only 4 pairs of residues (Thr23—Thr26, Ala295—Gln298, Lys628—Cys697, Ala97—Ala149) for NiV_BGD_V1 (Fig. 12) and only 1 pair (Gly69—Phe171) for NiV_BGD_V2 were selected for the disulfide bond formation because their energy is less than 2.2 and Chi3 value is between − 87 to + 97 (Table S6).

**Fig. 13:**
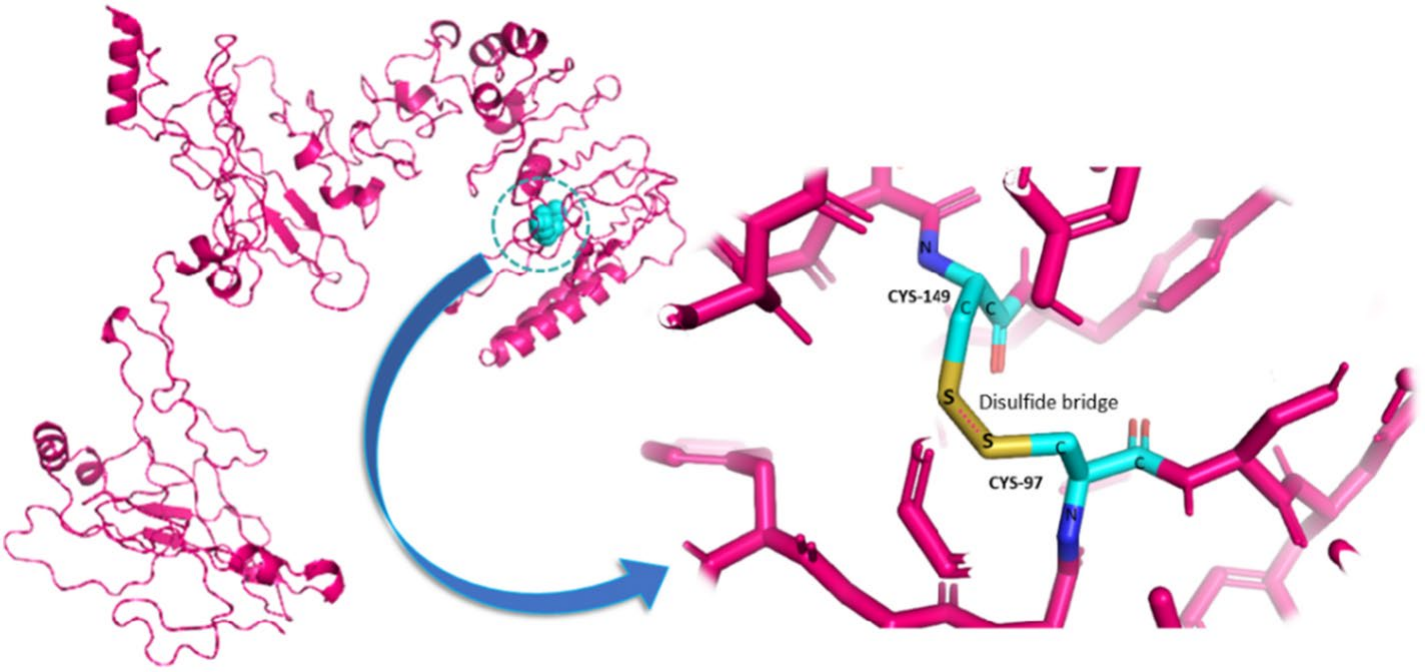
Three-dimensional view of the mutated vaccine candidate-1. The colored region depicts mutated Cys97 and Cys149 and a disulfide bond between them

The selected five pairs were evaluated through the DynaMut server to check the vaccine structure stability after the mutation. Only one pair (Ala97 and Ala149) of NiV_BGD_V1 among those residues fulfill a pair for the probable disulfide bond with stable mutation (ΔΔG = 1.113 kcal/mol and 1.430 kcal/mol) and decrease molecular flexibility (Fig. 13). Therefore, these two residues were taken into account for the mutation with cysteine. As NiV_BGD_V2 vaccine candidate could not fulfill a pair, disulfide engineering was omitted from this vaccine candidate.

### Molecular Docking of NiV_BGD_V1 and NiV_BGD_V2

**Fig. 14:**
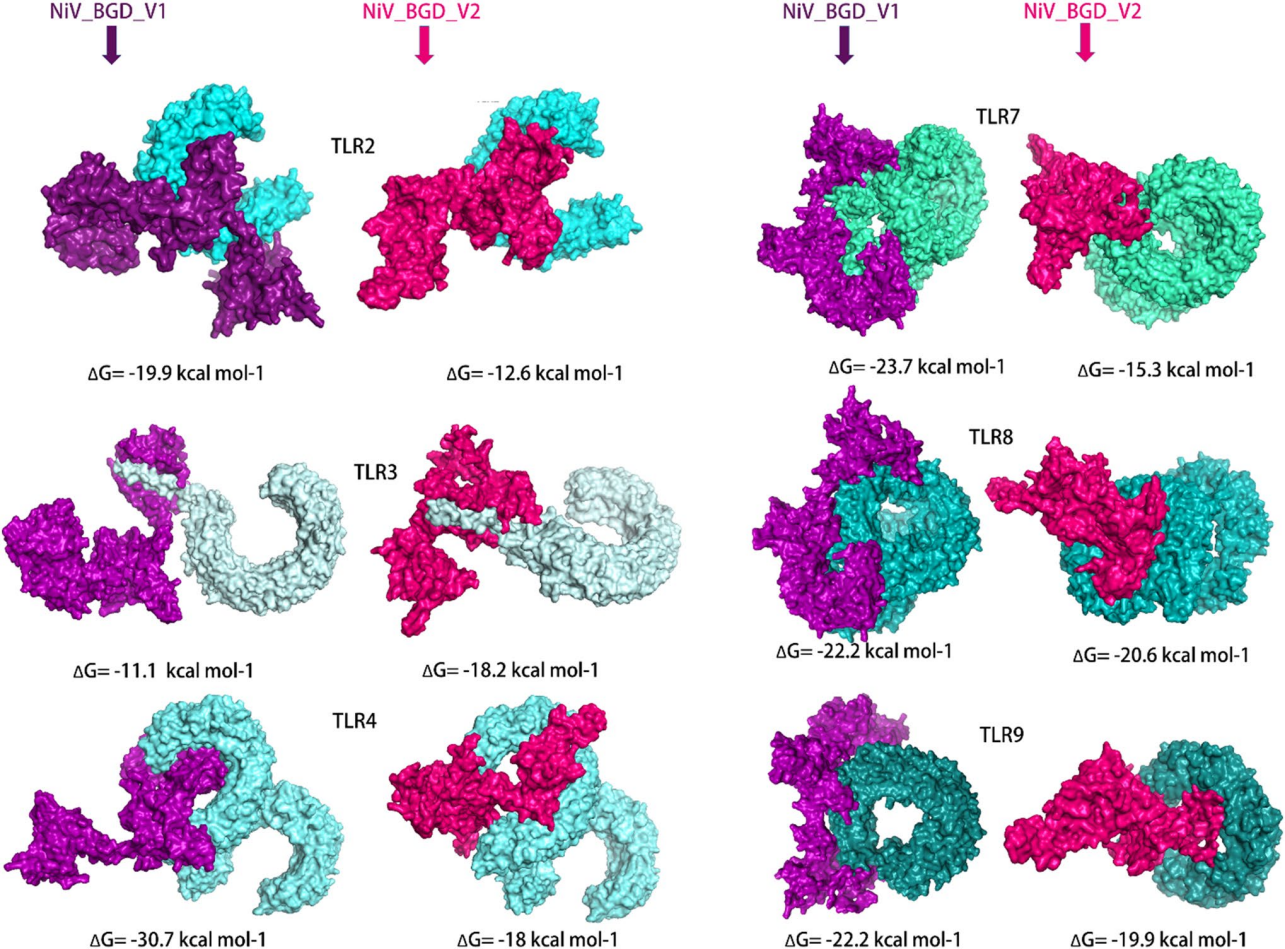
Molecular docking of NiV_BGD_V1 and NiV_BGD_V2 with virus specific TLRs. NiV_BGD_V1 and NiV_BGD_V2 vaccine protein represented by purple and magenta color,respectively. TLRs were represented by different shades of cyan color. Each binding affinity quantification in ΔG value is shown below each docking. Each docking resulted in negative ΔG value,denoting greater binding affinity

The immune response of TLRs (TLR2, TLR3, TLR4, TLR7, TLR8, TLR9) against NiV vaccine candidates (NiV_BGD_V1 and NiV_BGD_V2) were predicted using the ClusPro webserver (Fig. 14). TLRs are an important part of the antigen-specific immune response, resulting in acquired immunity (Takeda and Akira 2005). The negative ΔG value for each docking complex, estimated by the PRODIGY webserver, indicated the strong binding affinity of the vaccine candidates with virus-specific TLRs. As the lowest ΔG value indicates the highest binding affinity, NiV_BGD_V1 showed the strongest affinity with TLR4 (ΔG = − 30.7 kcal/mol, Kd (M) at 25.0 °C = 3.00E−23) while NiV_BGD_V2 had greater binding with TLR8 (ΔG = − 20.6 kcal/mol, Kd (M) at 25.0 °C  = 7.40E−16) (Table S7). Both vaccine candidates showed favorable interaction with NiV specific TLR3 (Basler 2012) with ΔG values − 11.1 kcal/mol and − 18.2 kcal/mol, respectively.

### Molecular Dynamic Simulation

**Fig. 15:**
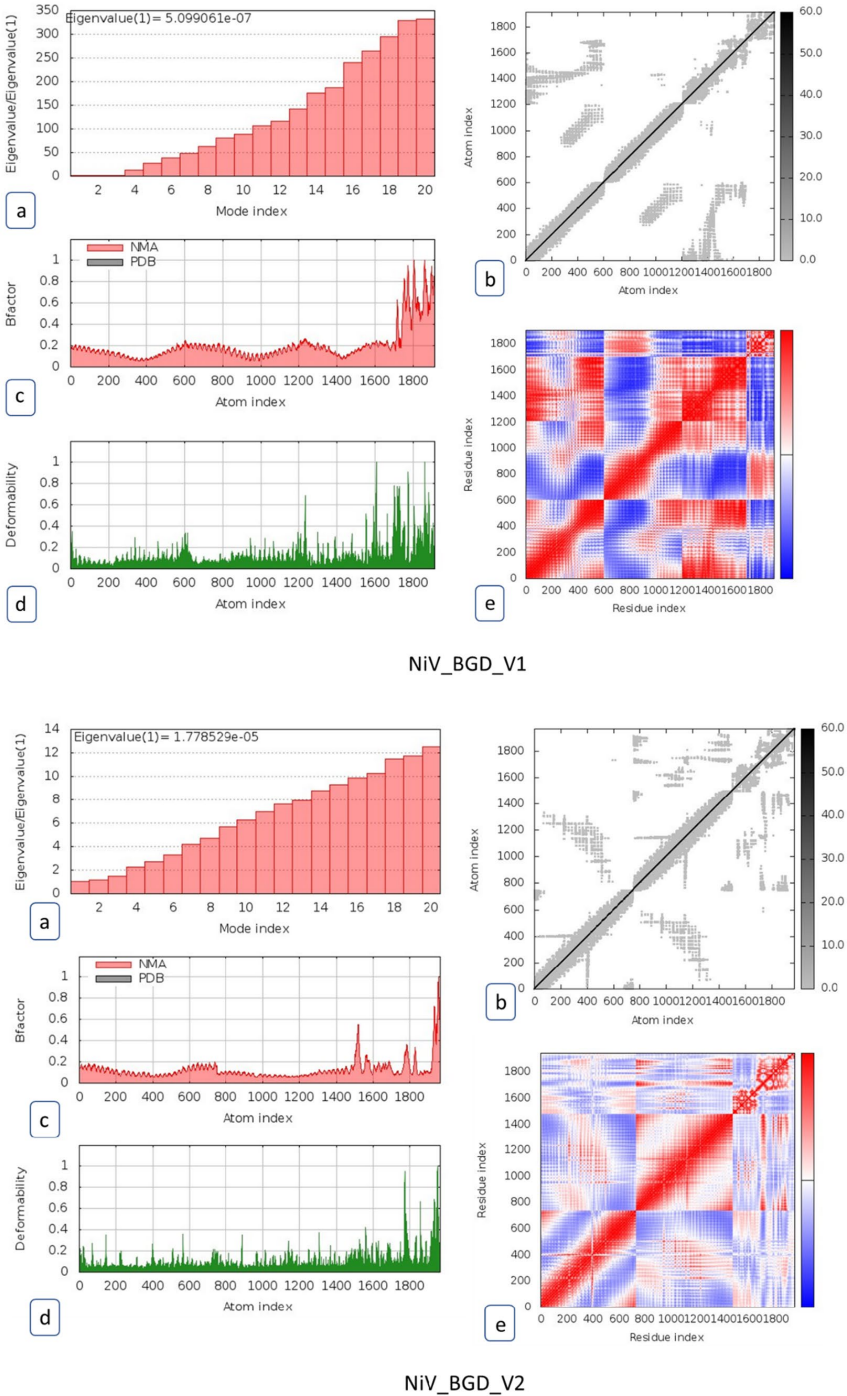
Molecular dynamic simulation of the vaccine candidates (NiV_BGD_V1-TLR4 and NiV_BGD_V2-TLR8). **a** Eigenvalue of the vaccine-receptor complex, **b** Elasticity network mapping, **c** Prediction of Bi-factor, **d** Co-variance map, **e** Main chain deformability

The iMod server predicted various dynamics state of the vaccine candidates. The dynamics are observed for NiV_BGD_V1-TLR4, and NiV_BGD_V2-TLR8 complexes as these vaccine candidates show the highest binding affinity with their respective bound toll-like receptors according to PRODIGY (PROtein binDIng enerGY prediction) (Honorato et al. 2021) webserver (Fig. 15). The deformability graphs show the presence of a coiled structure in the vaccine, which indicates the flexibility in the structure. The eigenvalue of NiV_BGD_V1-TLR4 is 5.099 × 10^–7^ and NiV_BGD_V2-TLR8 is 1.779 × 10^–5^, which indicates a lower amount of energy is required to deform the structure for both vaccine candidates with their respective TLRs. Elasticity mapping shows the flexibility of the vaccine candidates in the coiled region.

### Immune Simulation

**Fig. 16:**
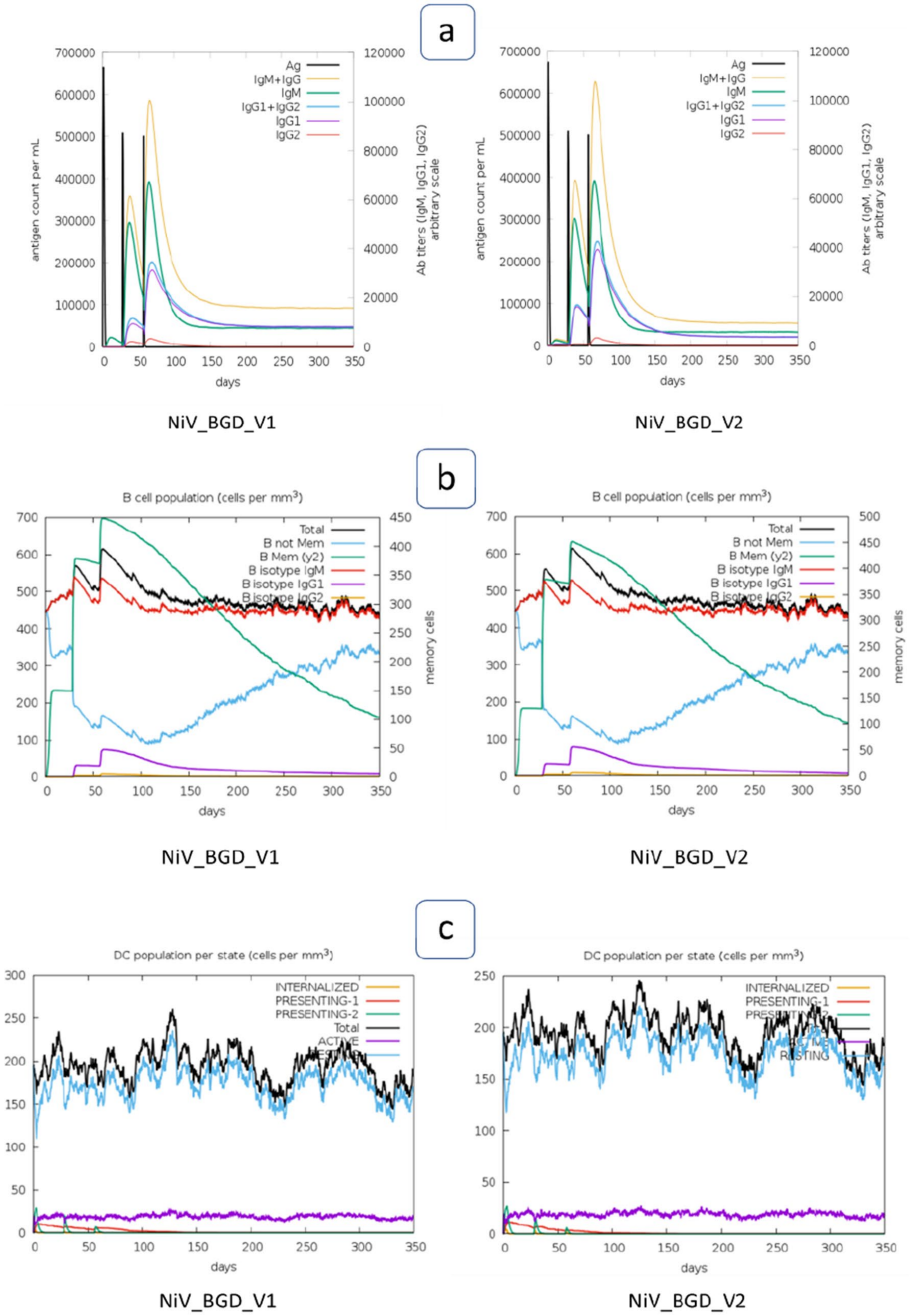

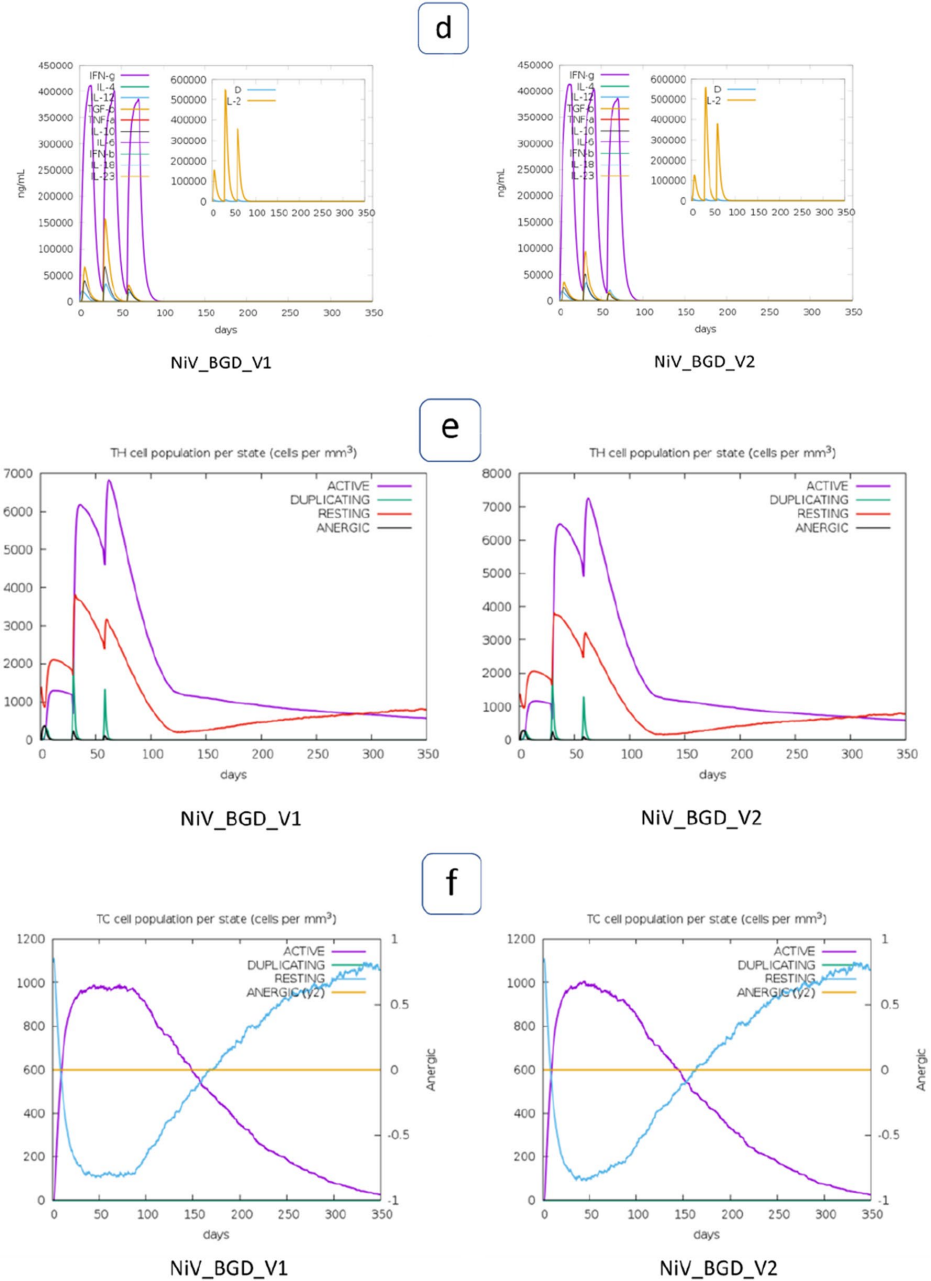
Simulation of the immune response of the vaccine candidates (NiV_BGD_V1 and NiV_BGD_V2). Both vaccine candidates are shown as antigens. **a** Immunoglobulin production after a vaccine injection, black line marks the presence of antigen. **b** Population of B-cell after subsequent exposure to antigen, **c** Dendritic cell population per state during one year after the initial injection, **d** Interferon response during one year window of vaccination, Simpson index is shown in the subset, **e** Helper T-cell population, **f** Cytotoxic T-cell level during one year after initial antigen exposure

Both vaccine candidates (NiV_BGD_V1 and NiV_BGD_V2) were uploaded onto the C-immsim server to simulate their immunological response for one year after administering a subject. Both vaccine candidates show a similar response after administration. Each vaccine administration shows a rise in the antigen level, which drops down significantly with time, while immunoglobulin levels show a steep increase (Fig. 16a). Different long-lasting B-cell isotypes were observed, indicating memory B-cell formation and subsequent isotype switching (Fig. 16b). Also, a Higher resting dendritic cell population was seen throughout the window of one year after the initial injection of the vaccine (Fig. 16c). Additionally, a high level of IFN-γ was seen after subsequent administration of the vaccine, marking a low Simpson index (D) (Fig. 16d). Furthermore, helper T-cell and cytotoxic T-cell counts were observed. Helper T-cell count increased with every injection, and gradually active cells decreased while resting helper T-cell count elevated (Fig. 16e). Active cytotoxic T-cell level showed a gradual decline during the window, and subsequently, resting cytotoxic T-cell level increased (Fig. 16f).

### Expression Prediction and In Silico Cloning of NiV_BGD_V1 and NiV_BGD_V2

**Fig. 17:**
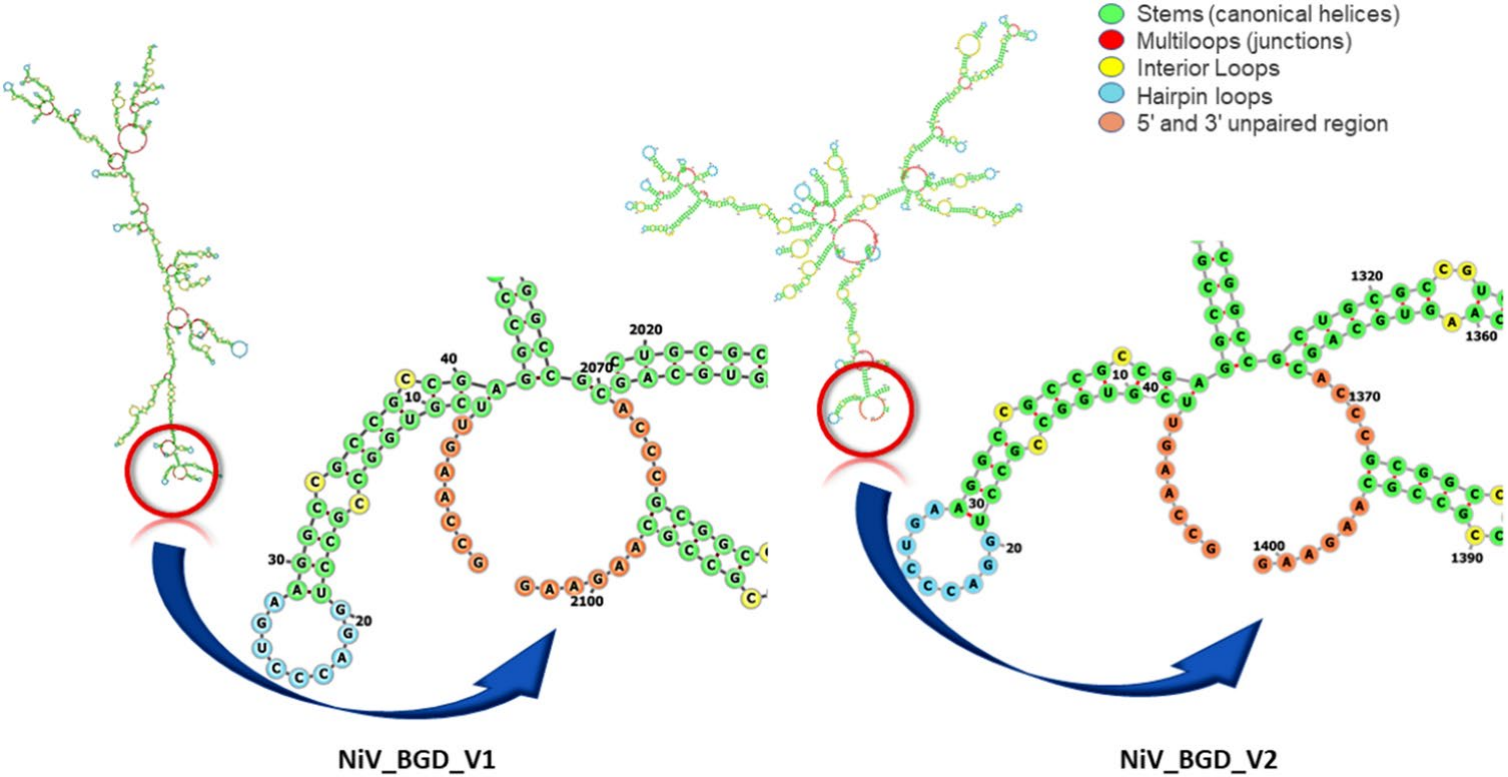
Secondary mRNA structure of A: NiV_BGD_V1 and B: NiV_BGD_V2. No pseudoknot formation is seen on the first 10 nucleotides in both candidate vaccines

The optimized codon sequence of NiV_BGD_V1 and NiV_BGD_V2 shows the Codon Adaptation Index (CAI) of 0.96 and 0.94. Moreover, the average GC content for these vaccine candidates was found to be 64.6% and 63.5%, respectively. A promising vaccine candidate should have a CAI value of 0.8–1.0 (with 1.0 indicating the highest degree of expression) and a GC content of 30–70 percent (Ali et al. 2017; Abdulla et al. 2019). These data suggest that each of our vaccine candidates has a good chance of improving human expression. Furthermore, the thermostability of the vaccine mRNA was demonstrated by the negative free energy of these vaccine designs, which were − 791.33 kcal/mol and − 545.23 kcal/mol, respectively, as determined by the 'RNAfold'. It is worth noting that the first 10 nucleotides of both chimeric mRNAs did not participate in stem formation, implying the absence of a pseudoknot or a persistent long hairpin structure (Fig. 17). Therefore, the host may easily commence the translation process since the ribosome's binding to the initiation site would not be disrupted.

**Fig. 18:**
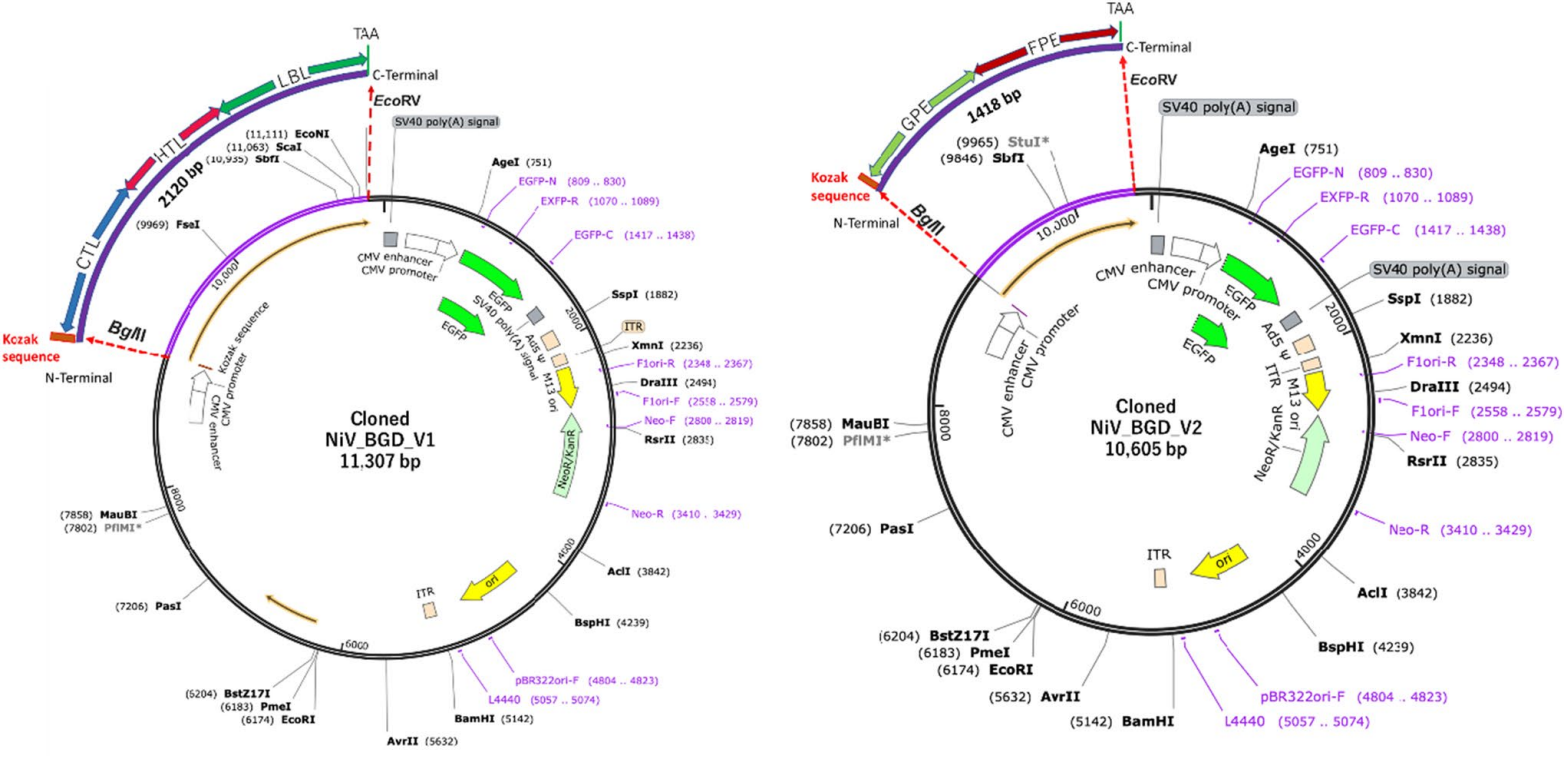
Cloned multi-epitope vaccine candidates into the pAdTrack-CMV expression vector

The optimized nucleotide sequences of NiV_BGD_V1 and NiV_BGD_V2 with added upstream Kozak sequence and downstream stop codon were incorporated into the pAdTrack-CMV vector under an inbuilt strong CMV promoter for the production of high-level recombinant protein (Wang et al. 2017). The final construct of cloned NiV_BGD_V1 and NiV_BGD_V2 containing recombinant plasmid was found to be 11,307 bp and 10,605 bp long (Fig. 18).

## Discussion

Due to the unavailability of a lisenced vaccine or drug to combat NiV infection in an individual, the battle is often one-sided, and Nipah virus (NiV) infections have always been devastating. Immunoinformatics analyses have been carried out looking for a suitable subunit vaccine against NiV infections. Many recent bioinformatics analyses proposed vaccine candidate designs depend on building epitopes from a specific protein. On the other hand, NiV is an RNA virus prone to spontaneous mutations that may accelerate escape mutation to overcome immune selection due to vaccine incorporation if the vaccine is designed to target a single antigen. The approach of this work was based on designing dual antigenic multi-epitope (DAME)-based subunit vaccines against NiV infections. The advantages of designing DAME-based subunit vaccine/s are that the vaccine may contribute to a more robust and broader immunogenic response against wider variants of the viruses. Along with it, the multi-epitope vaccine has been proved to be safer with more logistical feasibility (Vartak and Sucheck 2016).

The study was initially planned with three different NiV proteins: G, F, and M. Since M protein has less accessibility on the surface which will render poor immunogenic responses as compared to G and F protein, even though M protein was predicted to be antigenic, excluded from further analysis. Only the surface accessible region of G and F protein was considered for epitope designing. Recent works in NiV attachment Glycoprotein (G protein) showed that the head domain of G protein is the main region that can elicit serum neutralizing activity upon administration of a vaccine that targets G protein. *Rhesus macaques* that have vaccinated with tetrameric NiV G ectodomains have shown the presence of neutralizing antibodies specific to the head region of G protein (Wang et al. 2022). Moreover, G and F proteins have been found to be the target of humoral immune responses in animals infected with NiV (Xu et al. 2013; Avanzato et al. 2019; Dang et al. 2019; Dang et al. 2021).

Using various servers, B-cell, CTL, and HTL epitopes were identified for G and F proteins. The epitopes were analyzed based on antigenicity, allergenicity, homology, and toxicity. Furthermore, HTL epitopes that can also invoke cytokines such as interferon-gamma (IFN-γ), interleukin—4 (IL-4), interleukin—10 (IL-10) were chosen. Cytokines can work as an important mediator for protection. CTL and HTL epitopes were analyzed based on docking with their predicted alleles of MHC-I and MHC-II. Representative epitopes showed effective binding with their respective alleles where the ΔG score was significantly lower, predicting a strong CTL and HTL response.

Both CTL and HTL epitopes of the proposed NiV-vaccines covered most of the global population but combinedly covered 99.99%. Population coverage around the region where the NiV outbreak was previously observed was also satisfactory. The epitopes were then merged for vaccine designings into two different manners. In Design-1, linkers were added between the epitopes for minimizing junctional immunogenicity. In contrast, in Design-2, instead of linkers between the epitopes, a chimeric vaccine with the junctional region between the dual antigens was designed to assess immunogenic responses that might be similar to natural infections. Adjuvants were used in both designs for a higher level of antigenic response. Using 3 different adjuvants (TLR4, β defensin, and Ribosomal protein L7/L12) and modifying the configuration of linker position in separate models, 12 vaccine sequence was constructed for further analysis.

Different webservers were used to determine antigenicity, allergenicity, and physicochemical properties. Solubility was also measured as insoluble protein vaccine in water will not be homogeneous in content. Physicochemical values indicated that selected vaccine candidates would be thermostable. Validity measurement eliminated 3 models of the vaccine as they were predicted to show allergenic response upon administration. The remaining nine models were considered for further validation in the following steps.

The secondary structure was identified to determine the proportions of alpha-helix, beta-sheet, coil, and turn. Most of the vaccine models have consisted predominantly of coils. The tertiary structure was determined using an online service to find out fragmentation in the 3-dimensional (3D) structure. The gap in the 3D structure will result in fragmentation of the protein and will make the protein subunit vaccine invalid/unstable. Of the remaining 9 models, 5 models showed no gap in their 3D structure resulting in a non-fragmented entity.

Ramachandran plot shows the stereochemical results of the protein residues. Two models showed the highest level of residues inside the allowed region and were chosen as vaccine candidates, while the other three models did not score up to the mark (> 80%) in Ramachandran plot analysis and were excluded from further evaluation. Vaccine candidate-1 (NiV_BGD_V1) showed 83.6%, and vaccine candidate-2 (NiV_BGD_V2) showed 89.3% in the allowed region with a minimal region in the unallowed region. Structure validation shows that the Z score of these two candidates is − 6.32 and − 6.67, respectively. The overall structure of the selected two vaccine candidates was found to be acceptable and well in range.

As 3D structure brings the different protein regions nearby, the closely brought region can act as a conformational epitope and elicit a B-cell response. Various webservers revealed that NiV_BGD_V1 and NiV_BGD_V2 resulted in five and eight epitopes, respectively. Disulfide engineering showed five probable pairs in close proximity in the proposed vaccine candidates to be able to mutate into cysteine. Through validation from the stereochemical standpoint, of the five probable pairs, only one pair found in vaccine candidate NiV_BGD_V1 (Ala97–Ala149) was capable of mutation into cysteine. Point to be noted that the predicted disulfide bond did not interfere with any epitopic region, which indicates no obstruction in vaccine outcome.

Molecular docking and molecular dynamic simulation were carried out between vaccine candidates and various toll-like receptors such as TLR2, TLR3, TLR4, TLR7, TLR8, and TLR9. Among the family of TLRs that are docked for binding, TLR2 and TLR9 are found to be effective in viral infections (Leoni et al. 2012; Martínez-Campos et al. 2017) while TLR3 is NiV specific to induce the antibody-mediated response (Basler 2012). TLR4, TLR7, and TLR8 can bind to RNA viruses (Heil et al. 2004; Brubaker et al. 2015). Therefore, docking was performed to identify the ability of the vaccine candidate to boost up innate immunity. These bindings showed negative ΔG values conveying the binding to be stable. NiV_BGD_V1 showed the highest binding affinity with TLR4, while NiV_BGD_V2 showed the highest binding affinity with TLR8. In the molecular dynamic simulation, NiV_BGD_V1-TLR4 and NiV_BGD_V2-TLR8 bindings were evaluated. In the case of both vaccines, the co-variance plot mostly shows a correlation between the residues. A lower eigenvalue suggests that less energy is needed to deform binding structures at different residues.

The immune simulation step was done to simulate the immune response when the vaccine is administered to a subject. In one year timeframe after the initial injection, the robust antibody response is seen in the case of both vaccine candidates. Both B-cell and Helper T-cell levels raise after each exposure and drop down with time while memory B-cell and resting helper T-cell formation takes place. Active Cytotoxic T-cell levels maintain a consistent level during the three injections but with time, active cell count drops give rise to resting CTLs. Cytokine levels increase with each vaccine exposure, which drops down gradually.

Codon adaptation was carried out to obtain a high level of expression of vaccine candidates when incorporated into a vector. NiV_BGD_V1 and NiV_BGD_V2 were analyzed for GC content (64.57 percent and 63.5 percent, respectively) and the codon adaptability index (0.96 and 0.94, respectively). Both parameters were favorable for high-level protein production in *Homo sapiens*. The secondary structure revealed no hairpin structure formation in the first 10 nucleotides in the vaccine mRNA sequence. The absence of pseudoknot indicated no inhibition of translation from the mRNA for production of the peptide vaccine would occur.

After successful gene cloning, the designed recombinant plasmid can be efficiently propagated using *E. coli* BJ5183 cells with an adenoviral backbone plasmid, such as pAdEasy-1. Multiple restriction endonuclease analyses can be performed to screen for recombinants of interest, such as kanamycin-resistant recombinants. Finally, the linearized recombinant plasmid will be transfected into adenovirus packagings cell lines, such as 911- or 293-cells, and recombinant adenoviruses will be produced within 7–10 days (He et al. 1998). Both adenovirus-based subunit vaccine candidates, NiV_BGD_V1 and NiV_BGD_V2, were predicted to have high levels of heterologous expression inside the human body after codon optimization. To facilitate the in vivo expression of stable mRNA, we incorporated the Kozak consensus sequence upstream of the optimized cDNA to ensure translation initiation from the genetic message and mediate ribosome assembly.

Virus-like particles (VLPs), DNA, and mRNA-based vaccines have gained increased popularity as potential vaccine candidates for various diseases. These vaccines can be expressed in mammalian and non-mammalian expression systems (bacteria, yeast, fungi). These expression systems have both advantages and disadvantages. While non-mammalian cells support faster production due to higher growth rates, mammalian cell lines generally provide properly folded vaccines with accurate post-translational modification. Virus-like particles are attractive to use as a robust vaccine. However, their limitations include low production yield along with high manufacturing costs. In contrast, Production costs are generally lower for adenoviral-based vaccines, and they can impart both cellular and humoral immunity (Chang 2021). Moreover, in clinical trials, adenovirus-based vaccines showed promising outcomes against various infectious diseases, including Malaria, Hepatitis C, Ebola, HIV, Tuberculosis, and Rotavirus, as well as cancers such as lymphoma, melanoma, prostate cancer, and others (Cai et al. 2020; Khan et al. 2021). Several Adenovirus-based COVID-19 vaccines have already been approved for human use, including the University of Oxford and AstraZeneca's ChAdOx1 nCoV-19 or AZD1222, Johnson & Johnson’s AD26.COV.2.S or JNJ-78436735, Gamaleya's Sputnik V, and CanSino Biologics' Ad5-nCoV (Khan et al. 2021). The findings of this study proposed an adenovirus-backboned vaccine based on the immunoinformatics approach. However, more robust wet lab-based animal studies will be essential prior to the implementation of human clinical trials.

## Conclusions

Given the highly pathogenic characteristics of NiV, its pandemic potential, and the lack of availability of approved therapeutics for treatments (i.e., monoclonal antibodies, small molecular drugs), it is necessary to develop a safe and effective vaccine against NiV. This study used an immunoinformatics approach to predict effective dual antigenic multi-epitope chimeric subunit vaccines capable of escalating a strong immune response by triggering both humoral and cellular immunity. The vaccine designs effectively met the criteria for antigenicity, allergenicity, immunogenicity, physicochemical properties, and inducing the immune response without affecting host cell housekeeping functions. The proposed vaccine constructs in this study could be promising candidates for protective vaccination against NiV.

## Supplementary Information

Below is the link to the electronic supplementary material.

Supplementary file1 (DOCX 608 kb)

Supplementary file2 (DOCX 17 kb)

Supplementary file3 (DOCX 21 kb)

Supplementary file4 (DOCX 16 kb)

Supplementary file5 (DOCX 14 kb)

Supplementary file6 (DOCX 16 kb)

Supplementary file7 (DOCX 15 kb)

Supplementary file8 (DOCX 14 kb)

## Data Availability

The datasets generated or analysed during this study are included in this article and its supplementary information files.
