# Supplementary material for: An Immunoinformatics Prediction of Novel Multi-Epitope Vaccines Candidate Against Surface Antigens of Nipah Virus
Source: Int J Pept Res Ther. 2022 Jun 23;28(4):123. doi: 10.1007/s10989-022-10431-z (PMC9219388; doi:10.1007/s10989-022-10431-z)
Supplement: Supplementary file 1 — Supplementary file1 (DOCX 608 kb) [file 10989_2022_10431_MOESM1_ESM.docx]

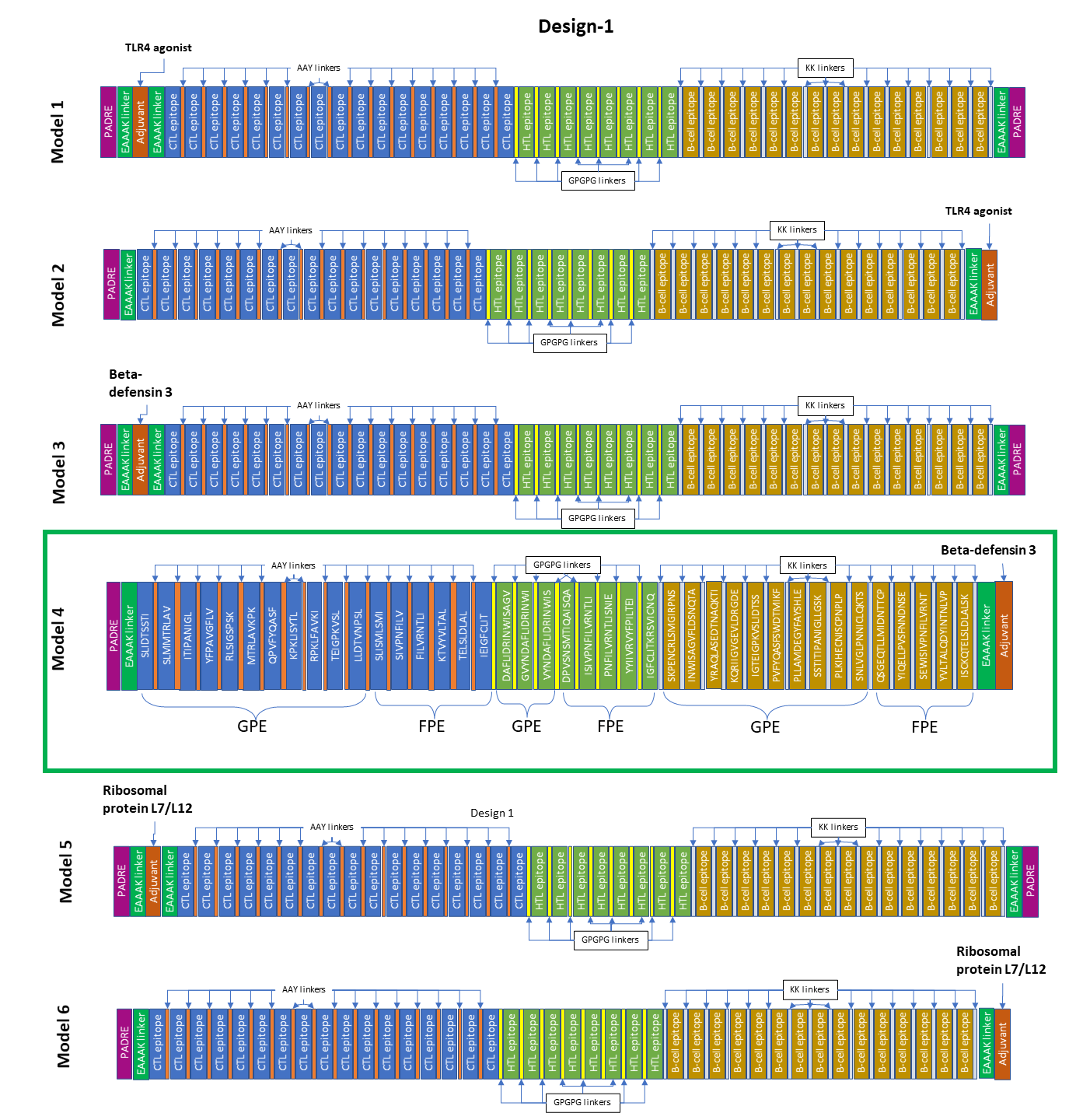


**Fig. S1-A: Vaccine construction of Design-1.** Total six models of Design-1 have been constructed by applying 3 different adjuvants and by varying number of PADRE sequence. All the CTL, HTL and LTL epitopes have been linked by respective linker (AAY, GPGPG and KK respectively). EAAAK linker was used between the conjunction of PADRE and adjuvant or PADRE and epitopes. Selected vaccine construction from Design-1 has been marked by green box. (GPE- G-proteins epitopes, FPE- F-proteins epitopes).


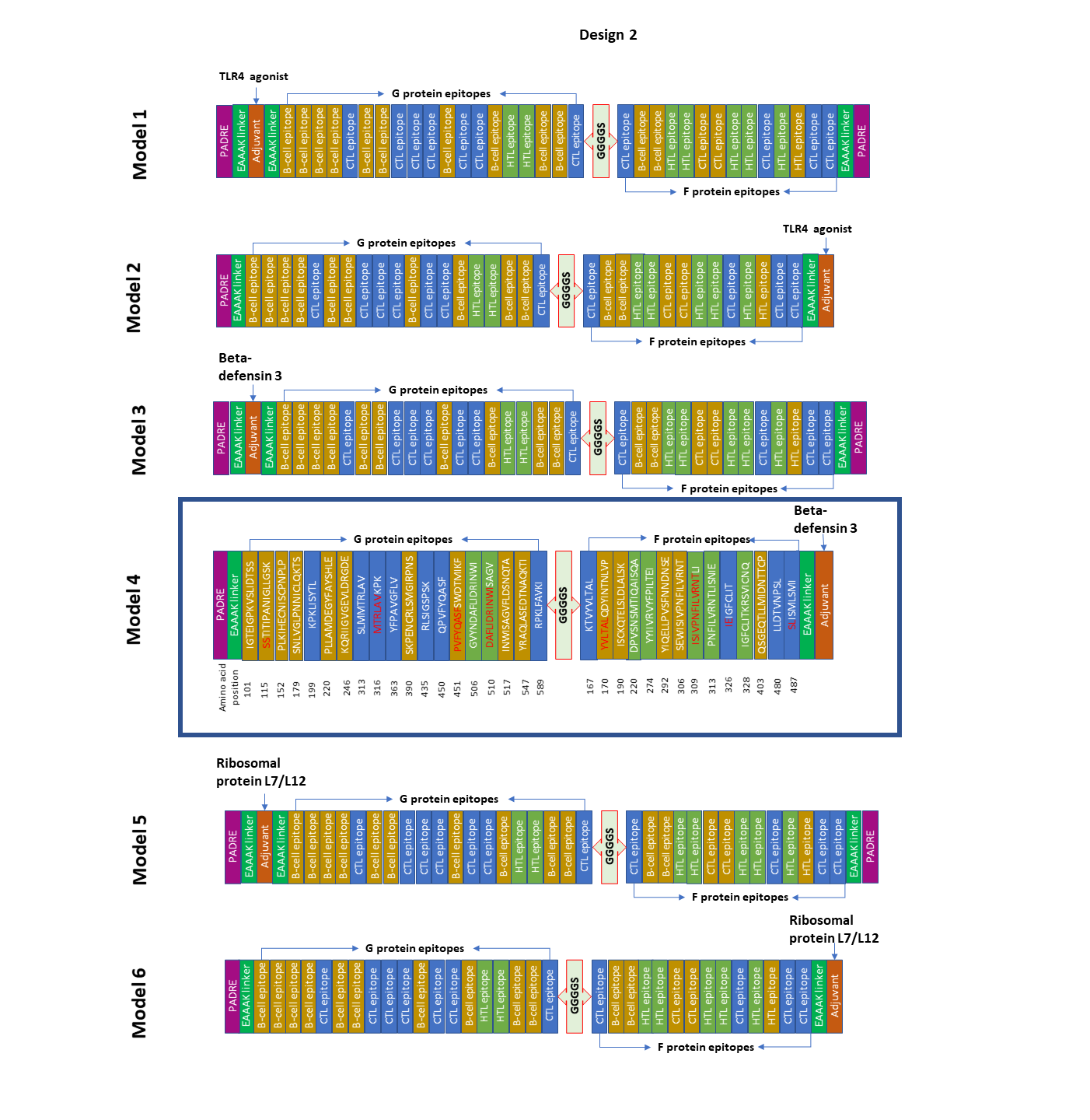


**Fig. S1-B: Vaccine construction of Design-2.** Six models of Design-2 have been constructed by applying the same adjuvant and PADRE variation used in Design-1. However, the main distinct property from Design-1 is the arrangement pattern of epitopes that are based on amino acid position and no linker was added in between the same protein epitopes. Most of the overlapping sequence was eliminated from the vaccine construct (marked red front). GPE and FPE has been linked together by the GGGGS linker to form a chimeric vaccine structure. The selected vaccine construction from Design-2 has been marked by a blue box. For both S1-A and S1-B, details representative sequence has been visualized only to the selected model.
