# Supplementary material for: An Immunoinformatics Prediction of Novel Multi-Epitope Vaccines Candidate Against Surface Antigens of Nipah Virus
Source: Int J Pept Res Ther. 2022 Jun 23;28(4):123. doi: 10.1007/s10989-022-10431-z (PMC9219388; doi:10.1007/s10989-022-10431-z)
Supplement: Supplementary file 2 — Supplementary file2 (DOCX 17 kb) [file 10989_2022_10431_MOESM2_ESM.docx]

| **Collection Date** | **Accession Number** | **G protein GenBank id** | **F protein GenBank id** | **Collection Date** | **Accession Number** | **G protein GenBank id** | **F protein GenBank id** |
| --- | --- | --- | --- | --- | --- | --- | --- |
| 2019 | MN549409 | QKV44068.1 | QKV44067.1 | 2011 | MK673573 | [QHR79082.1](https://www.ncbi.nlm.nih.gov/protein/QHR79082.1) | QHR79081.1 |
| 2018 | MH396625 | AWT50994.1 | AWT50993.1 | 2011 | MK673574 | QHR79091.1 | QHR79090.1 |
| 2018 | MH523640 | QBQ56705.1 | QBQ56704.1 | 2011 | MK673575 | [QHR79100.1](https://www.ncbi.nlm.nih.gov/protein/QHR79100.1) | QHR79099.1 |
| 2018 | MH523641 | QBQ56714.1 | QBQ56713.1 | 2011 | MK673576 | [QHR79109.1](https://www.ncbi.nlm.nih.gov/protein/QHR79109.1) | QHR79108.1 |
| 2018 | MH523642 | QBQ56723.1 | QBQ56722.1 | 2011 | MK673577 | [QHR79118.1](https://www.ncbi.nlm.nih.gov/protein/QHR79118.1) | QHR79117.1 |
| 2015 | MK673584 | QHR79181.1 | QHR79180.1 | 2011 | MK673578 | QHR79127.1 | QHR79126.1 |
| 2015 | MK673585 | QHR79190.1 | QHR79189.1 | 2008 | JN808857 | AEZ01382.1 | AEZ01381.1 |
| 2015 | MK673586 | QHR79199.1 | QHR79198.1 | 2008 | JN808863 | AEZ01389.1 | AEZ01388.1 |
| 2015 | MK673587 | QHR79208.1 | QHR79207.1 | 2008 | MK673568 | QHR79046.1 | QHR79045.1 |
| 2014 | MK673589 | QHR79226.1 | QHR79225.1 | 2007 | FJ513078 | [ACT32615.1](https://www.ncbi.nlm.nih.gov/protein/ACT32615.1) | ACT32614.1 |
| 2014 | MK673590 | QHR79235.1 | QHR79234.1 | 2004 | AY988601 | AAY43916.1 | AAY43915.1 |
| 2014 | MK673591 | QHR79244.1 | QHR79243.1 | 2004 | MK673564 | [QHR79010.1](https://www.ncbi.nlm.nih.gov/protein/QHR79010.1) | QHR79009.1 |
| 2014 | MK673592 | QHR79253.1 | QHR79252.1 | 2004 | MK673565 | [QHR79019.1](https://www.ncbi.nlm.nih.gov/protein/QHR79019.1) | QHR79018.1 |
| 2013 | MK575060 | QCY59033.1 | QCY59032.1 | 2004 | MK673566 | [QHR79028.1](https://www.ncbi.nlm.nih.gov/protein/QHR79028.1) | QHR79027.1 |
| 2013 | MK575061 | QCY59039.1 | QCY59038.1 | 2004 | MK673567 | [QHR79037.1](https://www.ncbi.nlm.nih.gov/protein/QHR79037.1) | QHR79036.1 |
| 2013 | MK575062 | QCY59045.1 | QCY59044.1 | 2003 | MK801755 | QDJ04463.1 | QDJ04462.1 |
| 2013 | MK575063 | QCY59050.1 | QCY59049.1 | 1999 | AF212302 | [AAK29088.1](https://www.ncbi.nlm.nih.gov/protein/AAK29088.1) | AAK29087.1 |
| 2013 | MK575064 | QCY59056.1 | QCY59055.1 | 1999 | AJ564621 | [CAD92351.1](https://www.ncbi.nlm.nih.gov/protein/CAD92351.1) | CAD92350.1 |
| 2013 | MK575065 | QCY59062.1 | QCY59061.1 | 1999 | AJ564622 | [CAD92357.1](https://www.ncbi.nlm.nih.gov/protein/CAD92357.1) | CAD92356.1 |
| 2013 | MK575066 | QCY59068.1 | QCY59067.1 | 1999 | AJ564623 | [CAD92363.1](https://www.ncbi.nlm.nih.gov/protein/CAD92363.1) | CAD92362.1 |
| 2013 | MK575067 | QCY59074.1 | QCY59073.1 | 1999 | AJ627196 | [CAF25497.1](https://www.ncbi.nlm.nih.gov/protein/CAF25497.1) | CAF25496.1 |
| 2013 | MK575068 | QCY59080.1 | QCY59079.1 | 1999 | AY029767 | [AAK50545.1](https://www.ncbi.nlm.nih.gov/protein/AAK50545.1) | AAK50544.1 |
| 2013 | MK575069 | QCY59086.1 | QCY59085.1 | 1999 | AY029768 | [AAK50554.1](https://www.ncbi.nlm.nih.gov/protein/AAK50554.1) | AAK50553.1 |
| 2013 | MK575070 | QCY59092.1 | QCY59091.1 | 1999 | MK673558 | [QHR78956.1](https://www.ncbi.nlm.nih.gov/protein/QHR78956.1) | QHR78955.1 |
| 2013 | MK673583 | QHR79172.1 | QHR79171.1 | 1999 | MK673559 | QHR78965.1 | QHR78964.1 |
| 2012 | MK673579 | QHR79136.1 | QHR79135.1 | 1999 | MK673560 | [QHR78974.1](https://www.ncbi.nlm.nih.gov/protein/QHR78974.1) | QHR78973.1 |
| 2012 | MK673581 | [QHR79154.1](https://www.ncbi.nlm.nih.gov/protein/QHR79154.1) | QHR79153.1 | 1999 | MK673561 | [QHR78983.1](https://www.ncbi.nlm.nih.gov/protein/QHR78983.1) | QHR78982.1 |
| 2012 | MK673582 | QHR79163.1 | QHR79162.1 | 1999 | MK673562 | [QHR78992.1](https://www.ncbi.nlm.nih.gov/protein/QHR78992.1) | QHR78991.1 |
| 2011 | MK673570 | QHR79055.1 | QHR79054.1 | 1999 | MK673563 | [QHR79001.1](https://www.ncbi.nlm.nih.gov/protein/QHR79001.1) | QHR79000.1 |
| 2011 | MK673571 | QHR79064.1 | QHR79063.1 | 1999 | NC_002728 | [NP_112027.1](https://www.ncbi.nlm.nih.gov/protein/NP_112027.1) | NP_112026.1 |

**Table S1: Accession number and GenBank id of retrieved 60 complete genome sequences of NiV G and F protein**
