# Supplementary material for: An Immunoinformatics Prediction of Novel Multi-Epitope Vaccines Candidate Against Surface Antigens of Nipah Virus
Source: Int J Pept Res Ther. 2022 Jun 23;28(4):123. doi: 10.1007/s10989-022-10431-z (PMC9219388; doi:10.1007/s10989-022-10431-z)
Supplement: Supplementary file 3 — Supplementary file3 (DOCX 21 kb) [file 10989_2022_10431_MOESM3_ESM.docx]

|  | **Epitopes** | **Supertype MHC alelle** | **Combined Score** | **Covered HLA types** |
| --- | --- | --- | --- | --- |
| **G protein** | SLIDTSSTI | 1.119 | A2 | HLA-A*02:01, HLA-A*02:02, HLA-A*02:03,HLA-A*02:04,HLA-A*02:05,HLA-A*02:06,HLA-A*02:07,HLA-A*68:02,HLA-A*69:01 |
|  | SLMMTRLAV | 1.0313 | A2 |  |
|  | ITIPANIGL | 0.7881 | A2 |  |
|  | YFPAVGFLV | 0.768 | A2 |  |
|  | RLSIGSPSK | 1.5554 | A3 | HLA-A*03:01,HLA-A*11:01,HLA-A*31:01,HLA-A*33:01,HLA-A*68:01 |
|  | MTRLAVKPK | 0.777 | A3 |  |
|  | QPVFYQASF | 1.4417 | B7 | HLA-B*07:02,HLA-B*35:01,HLA-B*35:02,HLA-B*35:03,HLA-B*51:01,HLA-B*51:02,HLA-B*51:03,HLA-B*51:04,HLA-B*51:05,HLA-B*51:06,HLA-B*51:07,HLA-B*51:08,HLA-B*51:09,HLA-B*51:10,HLA-B*51:12,HLA-B*51:13,HLA-B*51:14,HLA-B*51:15,HLA-B*51:18,HLA-B*51:21,HLA-B*51:22,HLA-B*51:27,HLA-B*51:31,HLA-B*51:32,HLA-B*51:33,HLA-B*51:34,HLA-B*51:37,HLA-B*53:01,HLA-B*54:01 |
|  | KPKLISYTL | 1.4229 | B7 |  |
|  | RPKLFAVKI | 1.2856 | B7 |  |
|  | TEIGPKVSL | 1.5786 | B44 | HLA-B*37:01, HLA-B*40:01,HLA-B*40:06,HLA-B*44:02,HLA-B*44:03 |
| **F protein** | LLDTVNPSL | 1.361 | A2 | HLA-A*02:01,HLA-A*02:02,HLA-A*02:03,HLA-A*02:04,HLA-A*02:05,HLA-A*02:06,HLA-A*02:07,HLA-A*68:02,HLA-A*69:01 |
|  | SLISMLSMI | 1.092 | A2 |  |
|  | SIVPNFILV | 1.0732 | A2 |  |
|  | FILVRNTLI | 1.0225 | A2 |  |
|  | KTVYVLTAL | 0.8166 | A2 |  |
|  | TELSLDLAL | 1.5595 | B44 | HLA-B*37:01,HLA-B*40:01,HLA-B*40:06,HLA-B*44:02,HLA-B*44:03 |
|  | IEIGFCLIT | 0.7969 | B44 |  |

**Table S2-A: Potential Cytotoxic T-cell Lymphocyte epitopes and their allelic characteristic**

**Table S2-B: Allelic characteristics and various cytokine inducing scoring of predicted Helper T-Lymphocyte epitopes.** Selected epitopes were able to cover a wide range of HLA alleles and induce at least one type of mentioned cytokine (IL-4, IL-10, IFN-γ).

|  | **Epitopes** | **Alleles** | **IFN-** γ | **IL-4** | **IL-10** |
| --- | --- | --- | --- | --- | --- |
| **G protein** | DAFLIDRINWISAGV | HLA-DRB1*04:01,HLA-DRB1*03:01,HLA-DRB1*08:02,HLA-DRB1*13:02,HLA-DRB1*12:01,HLA-DQA1*01:01,HLA-DQB1*05:01,HLA-DPA1*03:01,HLA-DPB1*04:02 | 0.39 (Inducer) | 0.25 (Inducer) | 0.342 (Inducer) |
|  | GVYNDAFLIDRINWI | HLA-DRB1*04:01,HLA-DQA1*01:01,HLA-DQB1*05:01,HLA-DRB1*03:01,HLA-DRB1*13:02,HLA-DPA1*02:01,HLA-DPB1*01:01 | 0.189 (Inducer) | 0.23 (Inducer) | 0.346 (Inducer) |
|  | VYNDAFLIDRINWIS | HLA-DRB1*04:01,HLA-DQA1*01:01,HLA-DQB1*05:01,HLA-DRB1*03:01,HLA-DRB1*13:02,HLA-DPA1*03:01,HLA-DPB1*04:02,HLA-DPA1*01:03,HLA-DPB1*02:01 | 0.243 (Inducer) | 0.26 (Inducer) | 0.476 (Inducer) |
| **F protein** | DPVSNSMTIQAISQA | HLA-DQA1*01:02,HLA-DQB1*06:02,HLA-DQA1*03:01,HLA-DQB1*03:02 | -0.149 (non-inducer) | 0.41(Inducer) | 0.191 (non-inducer) |
|  | ISIVPNFILVRNTLI | HLA-DRB1*01:01,HLA-DRB1*07:01,HLA-DRB1*15:01,HLA-DRB1*04:01,HLA-DRB1*04:05,HLA-DRB1*13:02,HLA-DRB1*11:01,HLA-DPA1*03:01,HLA-DPB1*04:02,HLA-DRB1*03:01 | 6 (non-inducer) | -0.95 (non-inducer) | 0.495 (Inducer) |
|  | PNFILVRNTLISNIE | HLA-DPA1*03:01,HLA-DPB1*04:02,HLA-DRB1*01:01,HLA-DRB1*04:01,HLA-DRB1*08:02,HLA-DRB1*04:05,HLA-DRB1*07:01,HLA-DRB1*13:02,HLA-DRB1*15:01,HLA-DRB1*11:01 | 2 (non-inducer) | 0.26 (Inducer) | 0.734 (Inducer) |
|  | YYIIVRVYFPILTEI | HLA-DPA1*02:01,HLA-DPB1*01:01,HLA-DPA1*01:03,HLA-DPB1*02:01,HLA-DQA1*01:01,HLA-DQB1*05:01,HLA-DRB1*15:01,HLA-DPA1*02:01,HLA-DPB1*05:01,HLA-DPA1*03:01,HLA-DPB1*04:02,HLA-DRB1*11:01,HLA-DQA1*05:01,HLA-DQB1*02:01,HLA-DRB1*08:02 | 1 (non-inducer) | 1.44 (Inducer) | 0.499 (Inducer) |
|  | IGFCLITKRSVICNQ | HLA-DRB1*11:01,HLA-DRB1*07:01,HLA-DRB1*09:01 | -0.678 (non-inducer) | 0.04 (Inducer) | 0.513 (Inducer) |
