# Supplementary material for: An Immunoinformatics Prediction of Novel Multi-Epitope Vaccines Candidate Against Surface Antigens of Nipah Virus
Source: Int J Pept Res Ther. 2022 Jun 23;28(4):123. doi: 10.1007/s10989-022-10431-z (PMC9219388; doi:10.1007/s10989-022-10431-z)
Supplement: Supplementary file 4 — Supplementary file4 (DOCX 16 kb) [file 10989_2022_10431_MOESM4_ESM.docx]

| **Vaccine Design** | **Properties** | **Model 1** | **Model 2** | **Model 3** | **Model 4** | **Model 5** | **Model 6** |
| --- | --- | --- | --- | --- | --- | --- | --- |
|  |  | **TLR4 Adjuvant** | | **Beta defensin Adjuvant** | | **Ribosomal protein L7/L12 Adjuvant** | |
| **Design-1** | **Antigenicity** | 0.5979 **(Antigen)** | 0.5984 **(Antigen)** | 0.5935 **(Antigen)** | 0.5924 **(Antigen)** | 0.5650 **(Antigen)** | 0.5667 **(Antigen)** |
|  | **Allergenicity** | Allergen | Non-allergen | Non-allergen | Non-allergen | Non-allergen | Non-allergen |
|  | **Toxicity** | Non-toxic | Non-toxic | Non-toxic | Non-toxic | Non-toxic | Non-toxic |
|  | **Solubility** | 0.454 | 0.439 | 0.437 | 0.424 | 0.448 | 0.432 |
|  | **Instability index** | 27.68 | 28.22 | 28.54 | 29.35 | 26.28 | 26.95 |
|  | **Aliphatic index** | 102.1 | 102.49 | 100.08 | 100.4 | 102.52 | 102.86 |
|  | **Grand Average of Hydropathicity (GRAVY)** | 0.138 | 0.127 | 0.087 | 0.074 | 0.146 | 0.136 |
| **Design-2** | **Antigenicity** | 0.6851 **(Antigen)** | 0.6894 **(Antigen)** | 0.6731 **(Antigen)** | 0.6729 **(Antigen)** | 0.6196 **(Antigen)** | 0.6238 **(Antigen)** |
|  | **Allergenicity** | Non-Allergen | **Allergen** | Non-Allergen | Non-allergen | Non-allergen | **Allergen** |
|  | **Toxicity** | Non-toxic | Non-toxic | Non-toxic | Non-toxic | Non-toxic | Non-toxic |
|  | **Solubility** | 0.25 | 0.255 | 0.32 | 0.304 | 0.397 | 0.384 |
|  | **Instability index** | 37.7 | 38.96 | 38.19 | 39.78 | 33.56 | 34.75 |
|  | **Aliphatic index** | 108.05 | 108.9 | 104.6 | 105.25 | 107.37 | 108.01 |
|  | **Grand Average of Hydropathicity (GRAVY)** | 0.233 | 0.219 | 0.149 | 0.133 | 0.223 | 0.212 |

**Table S3**: **Different** **property analysis of designed vaccine candidates.** Nine models out of 12 models were found to be non-allergenic and thus selected for further secondary and tertiary structure analysis. Design-1 Model-1, Design-2 Model-2, Design-2 Model-6 have been eliminated for predicted allergenicity.
