# Supplementary material for: An Immunoinformatics Prediction of Novel Multi-Epitope Vaccines Candidate Against Surface Antigens of Nipah Virus
Source: Int J Pept Res Ther. 2022 Jun 23;28(4):123. doi: 10.1007/s10989-022-10431-z (PMC9219388; doi:10.1007/s10989-022-10431-z)
Supplement: Supplementary file 5 — Supplementary file5 (DOCX 14 kb) [file 10989_2022_10431_MOESM5_ESM.docx]

**Table S4: Predicted secondary and tertiary structure properties of designed vaccine candidates**. The percentage of helix and coil structure has been predicted by using Self-Optimized Prediction method With Alignment (SOPMA) tool. ‘RaptorX’ server predict the tertiary structure and after visualization in PyMol, only 5 out of 9 models were selected further for their structural integrity.

| **Secondary structure properties** | **Design 1** | | | | | **Design 2** | | | |
| --- | --- | --- | --- | --- | --- | --- | --- | --- | --- |
|  | **Model 2** | **Model 3** | **Model 4** | **Model 5** | **Model 6** | **Model 1** | **Model 3** | **Model 4** | **Model 5** |
| **α-helix** | 30.47% | 32.55% | 30.53% | 38.93% | 37.66% | 32.87% | 23.92% | 19.70% | 34.21% |
| **β-sheet** | 27.75% | 26.56% | 27.25% | 24.00% | 24.55% | 24.01% | 30.72% | 31.48% | 26.49% |
| **Turn** | 8.90% | 8.62% | 8.70% | 8.71% | 8.91% | 7.69% | 8.04% | 7.71% | 8.25% |
| **Coil** | 32.88% | 32.27% | 33.52% | 28.36% | 28.88% | 35.43% | 37.32% | 41.11% | 31.05% |
| **Gap in Tertiary structure** | Gap | Gap | **No Gap** | **No Gap** | **No Gap** | Gap | Gap | **No Gap** | **No Gap** |
