# Supplementary material for: An Immunoinformatics Prediction of Novel Multi-Epitope Vaccines Candidate Against Surface Antigens of Nipah Virus
Source: Int J Pept Res Ther. 2022 Jun 23;28(4):123. doi: 10.1007/s10989-022-10431-z (PMC9219388; doi:10.1007/s10989-022-10431-z)
Supplement: Supplementary file 6 — Supplementary file6 (DOCX 16 kb) [file 10989_2022_10431_MOESM6_ESM.docx]

**Table S5-A: Predicted 8 Discontinuous Epitope(s) for NiV_BGD_V1**

| **No.** | **Residues** | **Number of residues** | **Score** |
| --- | --- | --- | --- |
| 1 | A:N316, A:T317, A:L318, A:I319, A:G320, A:P321, A:G322, A:P323, A:G324, A:P325, A:N326, A:F327, A:F353, A:P354, A:I355, A:L356, A:T357, A:E358, A:I359, A:G360, A:P361, A:G362, A:P363, A:G364, A:I365, A:G366, A:G406, A:V407, A:F408, A:L409, A:D410, A:S411, A:N412, A:Q413, A:T414, A:A415, A:K416, A:K417, A:Y418, A:R419, A:A420, A:Q421, A:L422, A:A423, A:S424, A:E425, A:D426, A:T427, A:N428, A:A429, A:Q430, A:K431, A:T432, A:I433, A:K434, A:K435, A:K436, A:Q437, A:R438, A:I439, A:I440, A:G441, A:V462, A:S463, A:L464, A:I465, A:D466, A:T467, A:S468, A:S469, A:K470, A:K471, A:P472, A:V473, A:F474, A:Y475 | 76 | 0.764 |
| 2 | A:E531, A:C532, A:N533, A:I534, A:S535, A:C536, A:P537, A:N538, A:P539, A:L540, A:P541, A:K542, A:K543, A:S544, A:N545, A:L546, A:V547, A:G548, A:L549, A:P550, A:N551, A:N552, A:I553, A:C554, A:L555, A:Q556, A:K557, A:T558, A:S559, A:K560, A:K561, A:Q562, A:S563, A:G564, A:E565, A:Q566, A:T567, A:L568, A:L569, A:M570, A:I571, A:D572, A:N573, A:T574, A:T575, A:C576, A:P577, A:K578, A:L584, A:L585, A:P586, A:V587, A:S588, A:F589, A:N590, A:N591, A:D592, A:N593, A:S594, A:E595, A:K596, A:K597, A:S598, A:E599, A:W600, A:I601, A:S602, A:V604, A:F607, A:I608, A:L609, A:V610, A:R611, A:N612, A:T613, A:K614, A:K615, A:Y616, A:V617, A:L618, A:T619, A:A620, A:L621, A:Q622, A:D623, A:Y624, A:I625, A:N626, A:T627, A:N628, A:L629, A:V630, A:P631, A:K632, A:K633, A:I634, A:S635, A:C636, A:K637, A:Q638, A:T639, A:E640, A:L641, A:S642, A:L643, A:D644, A:L645, A:A646, A:L647, A:S648, A:K649, A:K650, A:K651, A:E652, A:A653, A:A654, A:A655, A:K656, A:G657, A:I658, A:I659, A:N660, A:T661, A:L662, A:Q663, A:K664, A:Y666, A:C667, A:R668, A:V669, A:R670, A:G671, A:G672, A:R673, A:C674, A:A675, A:V676, A:L677, A:S678, A:C679, A:L680, A:P681, A:K682, A:E683, A:Q685, A:I686, A:G687, A:K688, A:C689, A:S690, A:T691, A:R692, A:G693, A:R694, A:K695, A:C696, A:C697, A:R698, A:R699 | 159 | 0.746 |
| 3 | A:A1, A:K2, A:F3, A:V4, A:A5, A:A6, A:W7, A:T8, A:L9, A:K10, A:A11, A:A12, A:A13, A:A15, A:A16, A:S19, A:L20, A:I21, A:D22, A:T23, A:T26, A:A29, A:Y30, A:L32, A:M33, A:M34, A:T35, A:R36, A:L37, A:A38, A:V39, A:A40, A:A41, A:Y42, A:I43, A:T44, A:I45, A:P46, A:A47, A:N48, A:I49, A:G50, A:L51, A:A52, A:A53, A:Y54, A:Y55, A:F56, A:P57, A:A58, A:V59, A:G60, A:F61, A:L62, A:V63, A:A64, A:A65, A:Y66, A:R67, A:L68, A:S69, A:I70, A:G71, A:S72, A:P73, A:S74, A:K75, A:A76, A:A77, A:Y78, A:M79, A:T80, A:F99, A:A100, A:A101, A:Y102, A:K103, A:P104, A:K105, A:L106, A:I107, A:S108, A:Y109, A:T110, A:L111, A:R115, A:F119, A:A120, A:V121, A:K122, A:I123, A:A124, A:A125, A:Y126, A:T127, A:E128, A:I129, A:G130, A:P131, A:K132, A:V133, A:S134, A:S163, A:I164, A:V165, A:P166, A:N167, A:F168, A:V171, A:T188, A:V189, A:Y190, A:V191, A:L192, A:T193, A:A194, A:E212, A:G214, A:F215, A:L217, A:I218, A:T219, A:G220 | 123 | 0.666 |
| 4 | A:P116, A:K117, A:L118, A:V143 | 4 | 0.595 |
| 5 | A:S202, A:L203, A:D204, A:L207 | 4 | 0.537 |

**Table S5-B: Predicted 8 Discontinuous Epitope(s) for NiV_BGD_V2**

| **No.** | **Residues** | **Number of residues** | **Score** |
| --- | --- | --- | --- |
| 1 | A:G437, A:G438, A:R439 | 3 | 0.939 |
| 2 | A:K422, A:N426, A:T427, A:Q429, A:K430, A:Y431, A:Y432, A:C433, A:R434, A:V435, A:R436, A:C440, A:A441, A:V442, A:L443, A:S444, A:C445, A:L446, A:P447, A:K448, A:E449, A:E450, A:Q451, A:I452, A:G453, A:K454, A:C455, A:S456, A:T457, A:R458, A:G459, A:R460, A:K461, A:C462, A:C463, A:R464, A:R465, A:K467 | 38 | 0.799 |
| 3 | A:N147, A:R155, A:P156, A:N157, A:S158 | 5 | 0.773 |
| 4 | A:L213, A:S215, A:T218, A:A219, A:Y220, A:R221, A:A222, A:Q223, A:L224, A:A225, A:S226, A:E227, A:D228, A:T229, A:N230, A:A231, A:Q232, A:K233, A:T234, A:I235, A:R236, A:P237, A:K238, A:L239, A:F240, A:A241, A:V242, A:K243, A:I244, A:P309, A:I310, A:L311, A:N345, A:T346, A:L347, A:I348, A:P349, A:N350, A:L368, A:T370, A:I371, A:G372, A:F373, A:C374, A:L375, A:I376, A:T377, A:K378, A:S380, A:V381, A:I382, A:C383, A:N384, A:Q385, A:Q386, A:S387, A:G388, A:E389, A:Q390, A:T391, A:L392, A:L393, A:M394, A:I395, A:D396, A:N397, A:T398, A:T399, A:C400, A:P401, A:L402, A:L403, A:D404, A:T405, A:V406, A:N407, A:P408, A:S409, A:L410, A:I411, A:S412, A:S415 | 82 | 0.705 |
| 5 | A:A1, A:K2, A:F3, A:V4, A:A5, A:A6, A:W7, A:T8, A:L9, A:K10, A:A11, A:A12, A:A13, A:E14, A:A15, A:A16, A:A17, A:K18, A:I19, A:G20, A:T21, A:E22, A:I23, A:G24, A:P25, A:K26, A:V27, A:S28, A:L29, A:I30, A:D31, A:T32, A:S33, A:S34, A:G43, A:L44, A:L45, A:G46, A:S47, A:K48, A:P49, A:K51, A:P60, A:N61, A:P62, A:L63, A:P64, A:S65, A:N66, A:L67, A:V68, A:G69, A:L70, A:P71, A:N72, A:I74, A:C75, A:T79, A:S80, A:K81, A:P82, A:S86, A:Y87, A:T88, A:L89, A:P90, A:L91, A:L92, A:A93, A:M94, A:F99, A:H103, A:E105, A:Q107, A:R108, A:I109, A:I110, A:G111, A:V112, A:G113, A:E114, A:V115, A:L116, A:D117, A:G119, A:D120, A:E121, A:S122, A:M124, A:M125, A:T126, A:R127, A:L128, A:A129, A:V130, A:K131, A:P132, A:K133, A:Y134, A:F135, A:P136, A:R159, A:L160, A:S161, A:I162, A:G163, A:S164, A:P165, A:S166, A:K167, A:Q168, A:P169, A:V170, A:F171 | 114 | 0.649 |
| 6 | A:Q273, A:L276, A:S277, A:D279, A:L280, A:A281, A:L282, A:S283, A:K284, A:D285, A:P286, A:N289, A:S290 | 13 | 0.629 |
| 7 | A:L150, A:S151, A:M152, A:G153, A:I154 | 5 | 0.607 |
| 8 | A:N325, A:N326, A:D327, A:N328, A:S329, A:E330, A:S331, A:E332, A:W333 | 9 | 0.543 |
