# Supplementary material for: An Immunoinformatics Prediction of Novel Multi-Epitope Vaccines Candidate Against Surface Antigens of Nipah Virus
Source: Int J Pept Res Ther. 2022 Jun 23;28(4):123. doi: 10.1007/s10989-022-10431-z (PMC9219388; doi:10.1007/s10989-022-10431-z)
Supplement: Supplementary file 7 — Supplementary file7 (DOCX 15 kb) [file 10989_2022_10431_MOESM7_ESM.docx]

**Figure S6:** **Mutational nature and entropy for di-sulfide bond formation in** **NiV_BGD_V1 and NiV_BGD_V2 vaccine candidates.** In NiV_BGD_V1, total 4 pairs and in NiV_BGD_V2 only 1 pair of amino acid (A.A) sequences that showed energy less than 2.2 and Chi^3^ value between -87 to +97 were initially selected to be mutated in cysteine for di-sulfide bond formation.

| **Pair** | **Chi^3^** | **Energy** | **A.A position** | **A.A sequence** | **ΔΔG (kcal/mol)** | **Mutation nature** | **Vibrational entropy (kcal.mol-^1^.K^-1^)** | **Molecular flexibility** | **A.A position** | **A.A sequence** | **ΔΔG (kcal/mol)** | **Mutation nature** | **Vibrational entropy (kcal.mol^-1^.K-^1)^** | **Molecular flexibility** |
| --- | --- | --- | --- | --- | --- | --- | --- | --- | --- | --- | --- | --- | --- | --- |
| **NiV_BGD_V1** | | | | | | | | | | | | | | |
| **1** | 100.64 | 0.89 | 23 | THR | 0.373 | Stabilizing | -0.096 | Decrease | 26 | THR | 0.206 | Stabilizing | 0.000 | Increase |
| **2** | -88.62 | 0.89 | 295 | ALA | 0.657 | Stabilizing | -0.182 | Decrease | 298 | GLN | 0.166 | Stabilizing | 0.674 | Increase |
| **3** | 107.54 | 1.46 | 682 | LYS | -0.408 | Destabilizing | 0.553 | Increase | 697 | CYS | N.A | N.A | N.A | N.A |
| **4** | -77.07 | 1.76 | 97 | ALA | 1.113 | Stabilizing | 0 | Decrease | 149 | ALA | 1.430 | Stabilizing | -0.756 | Decrease |
| **NiV_BGD_V2** | | | | | | | | | | | | | | |
| **1** | 72.53 | 1.91 | 69 | GLY | 0.806 | Stabilizing | -0.517 | Decrease | 171 | PHE | -0.695 | Destabilizing | 0.571 | Increase |
