# Supplementary material for: An Immunoinformatics Prediction of Novel Multi-Epitope Vaccines Candidate Against Surface Antigens of Nipah Virus
Source: Int J Pept Res Ther. 2022 Jun 23;28(4):123. doi: 10.1007/s10989-022-10431-z (PMC9219388; doi:10.1007/s10989-022-10431-z)
Supplement: Supplementary file 8 — Supplementary file8 (DOCX 14 kb) [file 10989_2022_10431_MOESM8_ESM.docx]

|  | **NiV_BGD_V1** | | **NiV_BGD_V2** | |
| --- | --- | --- | --- | --- |
| **Receptor** | **ΔG (kcal mol-1)** | **Kd (M) at 25.0 ℃** | **ΔG (kcal mol-1)** | **Kd (M) at 25.0 ℃** |
| **TLR2** | -19.9 | 2.40*10^-15^ | -12.6 | 6.00*10^-10^ |
| **TLR3** | -11.1 | 7.40*10^-09^ | -18.2 | 4.80*10^-14^ |
| **TLR4** | -30.7 | 3.00*10^-23^ | -18 | 6.30*10^-14^ |
| **TLR7** | -23.7 | 4.10*10^-18^ | -15.3 | 6.10*10^-12^ |
| **TLR8** | -22.2 | 5.60*10^-17^ | -20.6 | 7.40*10^-16^ |
| **TLR9** | -22.2 | 4.80*10^-17^ | -19.9 | 2.40*10^-15^ |

**Table S7: Binding score(ΔG) and dissociation constant (Kd) of selected vaccine candidates with TLRs**
